# Convergent synthetic methodology for the construction of self-adjuvanting lipopeptide vaccines using a novel carbohydrate scaffold

**DOI:** 10.3762/bjoc.10.181

**Published:** 2014-07-30

**Authors:** Vincent Fagan, Istvan Toth, Pavla Simerska

**Affiliations:** 1 The University of Queensland, School of Chemistry and Molecular Biosciences, Cooper Road, St. Lucia QLD 4072, Australia; 2 The University of Queensland, School of Pharmacy, Pharmacy Australia Centre of Excellence, Cornwall Street, Woolloongabba, QLD 4072, Australia

**Keywords:** amino acid lipidation, cycloadditon reaction, multivalent glycosystems, peptide vaccine, tetrapropargyl glucopyranose

## Abstract

A novel convergent synthetic strategy for the construction of multicomponent self-adjuvanting lipopeptide vaccines was developed. A tetraalkyne-functionalized glucose derivative and lipidated Fmoc-lysine were prepared by novel efficient and convenient syntheses. The carbohydrate building block was coupled to the self-adjuvanting lipidic moiety (three lipidated Fmoc-lysines) on solid support. Four copies of a group A streptococcal B cell epitope (J8) were then conjugated to the glyco-lipopeptide using a copper-catalyzed cycloaddition reaction. The approach was elaborated by the preparation of a second vaccine candidate which incorporated an additional promiscuous T-helper epitope.

## Introduction

Vaccination is often the most effective and economic long-term way to prevent disease [1]. Through vaccination, successful outcomes have been achieved for diseases such as smallpox, polio and diphtheria, thus providing motivation for the development of superior vaccines for other diseases. Traditionally, vaccines consisted of killed or live attenuated pathogens, or their purified components. While these approaches have proved highly beneficial, in some instances they have shown associated risks, such as cross-reactivity with self-tissue (leading to autoimmune conditions), and the possibility of infection, particularly with immuno-compromized patients [2–3]. To overcome such issues, subunit vaccines, which contain only the minimum B and T cell peptide epitopes, were developed. However, when such peptide epitopes are administered alone, they are poorly immunogenic. This is due to their instability in the presence of proteases, and due to the absence of other important immuno-stimulatory components, known as adjuvants [4]. Adjuvant development has advanced slowly and for many decades, aluminium salts were the only adjuvants approved for clinical use [5]. Recently, research efforts have focused on the development of adjuvants which activate specific pattern recognition receptors (PRRs) found on immune cells. PRRs recognize conserved pathogen-associated molecular patterns. This leads to activation of cells of the innate and adaptive immune system, resulting in significantly enhanced immune responses [6]. By preparing agonists for a particular type of PRR (e.g., Toll Like Receptor (TLR)), an appropriate immune response can be induced for each type of pathogen [7]. The TLR2 is a receptor present on cells of the innate and adaptive immune system. It recognizes lipidic structural components of bacteria, fungi and viruses, and plays a key role in the body’s defences, particularly against Gram positive bacteria [8]. Research efforts within our group have shown that a short sequence of synthetic lipo-amino acids (Lipid Core Peptide (LCP)), can bind to the TLR2, which results in increased immune responses to otherwise poorly immunogenic peptide antigens [9–12].

The model pathogen chosen for this study was *Streptococcus pyogenes*, a Gram positive bacteria that affects the skin and upper respiratory tract, and causes a range of health issues that are collectively referred to as group A streptococcal (GAS) infections [13]. The most severe GAS related problems are post-streptococcal rheumatic fever and rheumatic heart disease, which are responsible for over half a million deaths annually [14]. Previously, we developed methodologies for the synthesis of carbohydrate building blocks as scaffold carriers of multiple B cell epitopes derived from GAS. The vaccine constructs consisted of the LCP adjuvanting moiety, and a carbohydrate core bearing four copies of a GAS B cell epitope [11,15–18]. When administered to B10.BR (H-2^k^) mice, the carbohydrate-based LCP vaccines elicited high serum IgG antibody titres [11]. One of the B cell epitopes used was the J8-peptide antigen. This epitope was identified from the conserved C-terminal region of the M protein (a cell surface protein and a major virulence factor) of *S. pyogenes* [19]. For comparison purposes, the J8 epitope was also employed in the current studies.

The previously reported carbohydrate-based vaccine constructs [11] were prepared by a divergent approach, where the carbohydrate core was coupled to the resin-bound LCP adjuvanting moiety, followed by stepwise synthesis of the B cell epitopes using solid-phase peptide synthesis (SPPS). Using this divergent approach, purification could not be performed until the end of the synthesis and crude samples of final vaccine candidates often required multiple purification steps. The current study outlines a new convergent synthetic methodology for the synthesis of novel carbohydrate-based GAS vaccine delivery systems, where each building block can be synthesized and purified prior to final assembly of the vaccine candidates. The building blocks may be assembled in different ways so that libraries of vaccine candidates can be prepared faster and more efficiently compared to the previous divergent approach.

## Results and Discussion

**Scheme 1:**
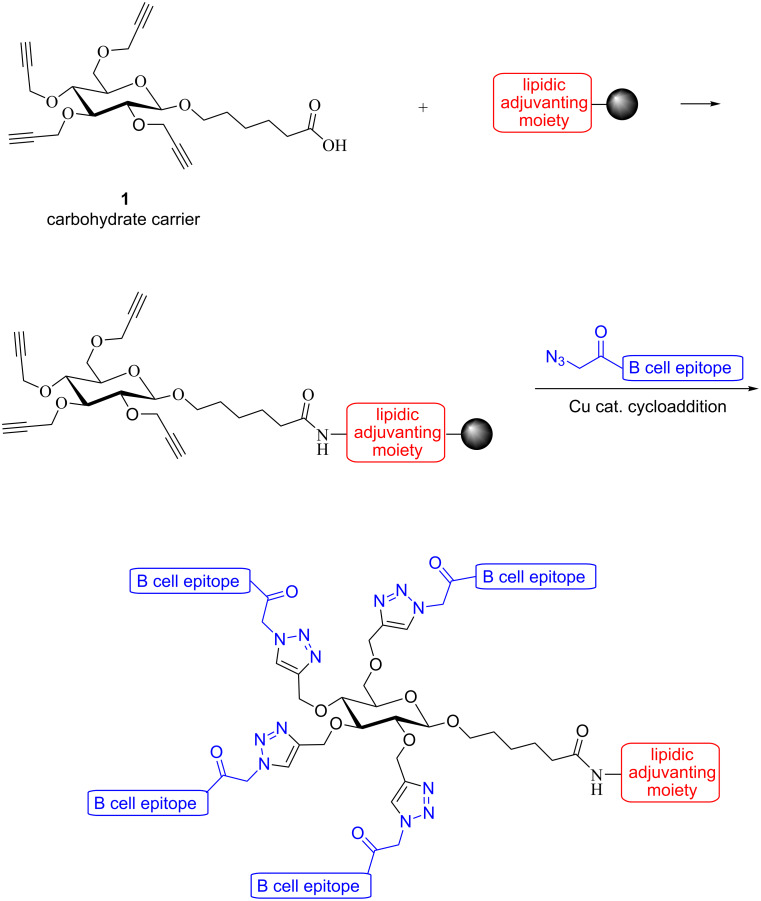
Convergent construction of self-adjuvanting vaccines bearing multiple copies of a B cell epitope.

As part of the new convergent approach, here, we report an efficient and convenient synthesis of a versatile alkyne-functionalized carbohydrate building block **1**, which can be conveniently incorporated into peptide sequences using SPPS (Scheme 1). The copper-catalyzed alkyne–azide cycloaddition ‘click’ reaction [20] was employed to couple multiple copies of purified B cell peptide antigens onto the carbohydrate core and lipidic adjuvanting moiety.

This type of convergent approach has a number of advantages over the previously described divergent route. Since each component is synthesized separately and purified before final assembly, purification of the vaccine candidates is less demanding. As a result, additional components can be incorporated, giving vaccine candidates of greater complexity. Taking advantage of this, we synthesized a second vaccine candidate containing a known promiscuous T-helper epitope [21], the lipidic adjuvanting moiety and four copies of the GAS B cell epitope coupled to the carbohydrate carrier. Although B cells may be activated via a number of T cell-independent routes, the T cell-dependent activation of B cells can lead to stronger and more long-term immune responses [6].

### Synthesis of carbohydrate carrier

**Scheme 2:**
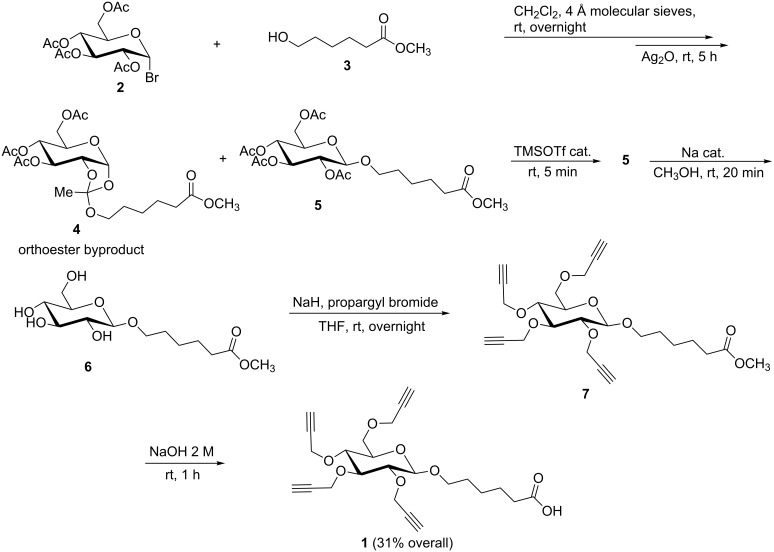
Synthesis of carbohydrate building block **1**.

Tetra-*O*-acetyl-α-D-glucopyranosyl bromide (**2**) was prepared according to a literature procedure [22] and immediately used in the proceeding Koenigs–Knorr glycosylation of methyl 6-hydroxyhexanoate (**3**). Hydroxy ester **3** was synthesized in high yield (81%) by addition of a catalytic amount of H_2_SO_4_ to ε-caprolactone in methanol, according to Duffy et al. [23]. However, the procedure was optimized by decreasing the reaction time from 48 h to 30 minutes and purification by distillation was not required. The glycosylation was carried out by addition of silver(I) oxide to a solution of glycosyl bromide **2** and hydroxy ester **3**, which yielded a mixture of orthoester byproduct **4** and desired glycosylation product **5** (Scheme 2). A catalytic amount of trimethylsilyl trifluoromethanesulfonate (TMSOTf) was added to the mixture, which resulted in ring-opening of orthoester **4** and conversion to the desired glycosylation product **5**. The presence of unreacted hydroxy ester **3** made purification of glycosylation product **5** difficult. Therefore, the crude reaction mixture was subjected to Zemplén deacetylation [24] to give intermediate **6**, and the unreacted hydroxy ester **3** was removed during work-up. The residue was alkylated with sodium hydride and propargyl bromide in dry tetrahydrofuran (THF) to give intermediate **7**, which was not isolated. The methoxy ester was hydrolyzed in situ by addition of sodium hydroxide. Acid–base extraction and purification by flash chromatography gave the novel carbohydrate building block **1** in 31% overall yield (Scheme 2). Similar carbohydrates have been used for the preparation of glycoclusters for lectin binding [25–28] and as scaffold carriers of multiple peptide antigens [17,29]. However, the synthesis of the new carbohydrate building block **1** reported here is concise and efficient, involving only one chromatographic step and takes advantage of the highly efficient “click” reaction.

### Synthesis of lipo-amino acid

**Scheme 3:**
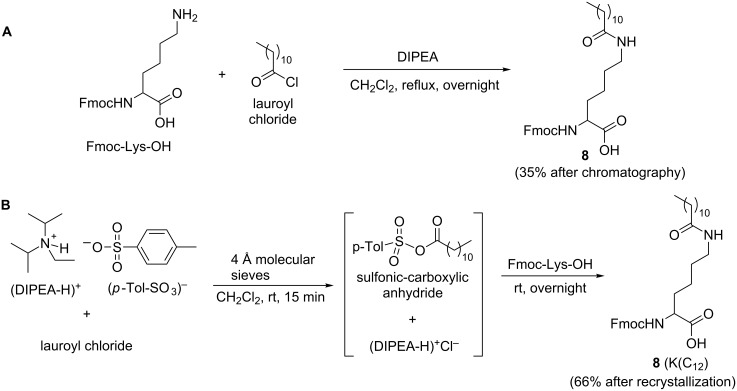
(A) Lipidation of Fmoc-Lys-OH with lauroyl chloride: (B) Lipidation of Fmoc-Lys-OH with sulfonic-carboxylic anhydride.

Lipidation of peptides is important as it is often used to improve stability and permeability of potential therapeutics [30]. The lipo-amino acids used previously for synthesis of the LCP system [31] were prepared by a multistep synthesis which resulted in a racemic mixture. In the current study, enantiomerically pure lipidated Fmoc-lysine (**8**) was synthesized and used to prepare the lipidic adjuvanting moiety. Treatment of commercially available Fmoc-Lys-OH with lauroyl chloride, in the presence of diisopropylethylamine (DIPEA), gave lipidated Fmoc-lysine (**8**) in 35% yield after flash chromatography (Scheme 3A). The low yield could be attributed to the formation of a significant amount of a dimeric lysine byproduct, which was not characterized. A number of different conditions were explored in order to inhibit the formation of the byproduct. By utilizing a sulfonic-carboxylic anhydride as the acylating agent, lipidated product **8** (K(C_12_)) could be synthesized smoothly and formation of the byproduct was avoided. The sulfonic-carboxylic anhydride was prepared in situ by the addition of lauroyl chloride to a solution containing DIPEA and *p*-toluenesulfonic acid, and Fmoc-Lys-OH was subsequently added to this mixture (Scheme 3B).

Because of this optimization, chromatography was not necessary and the lipidated product **8** could be purified by recrystallization (66% yield) (Scheme 3B). Compound **8** has been synthesized previously by Zhang et al. [32] using a procedure which required chromatography. The simple modification reported here significantly increased the efficiency of the procedure and may have wider application for the convenient acylation of Fmoc-lysine.

### Synthesis of azide functionalized J8 B cell epitope (N_3_-J8)

The conserved C-terminal region of the M protein adopts an α-helical secondary structure [13]. In order to maintain this native secondary structure, the J8 epitope (QAEDKVKQ-**SREAKKQVEKAL**-KQLEDKVQ) consists of a short C-terminal peptide from the M protein (in bold), placed within a sequence derived from the GCN4 leucine zipper DNA binding protein of yeast, which is known to promote an α-helical structure [33]. This chimeric peptide has been shown to elicit systemic IgG antibodies, which were capable of opsonizing the GAS pathogen and inducing protective immunity against GAS [19]. In the current study, the J8 epitope was synthesized using standard Fmoc SPPS. Azidoacetic acid was synthesized according to Brabez et al. [34] and was coupled to the N-terminus of the J8 epitope. The peptide was cleaved from the resin and purified by preparative RP (reversed phase)-HPLC.

### Construction of vaccine candidates

**Scheme 4:**
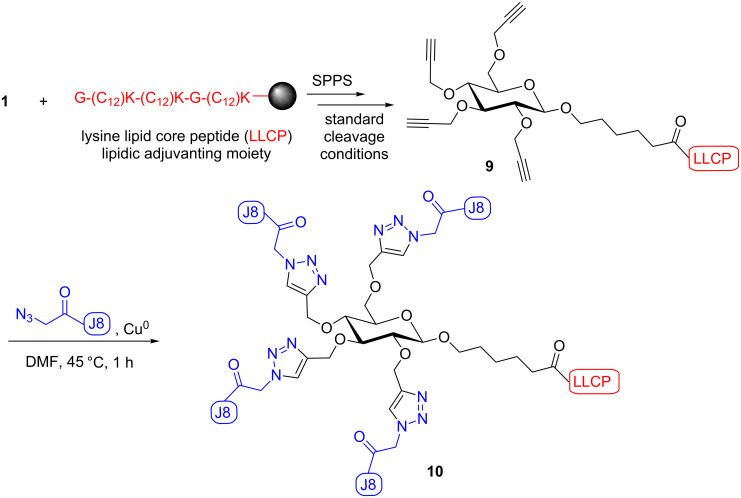
Convergent synthesis of self-adjuvanting vaccine candidate **10** consisting of lipidic adjuvanting moiety LLCP, carbohydrate carrier and four copies of J8 peptide epitope.

**Figure 1:**
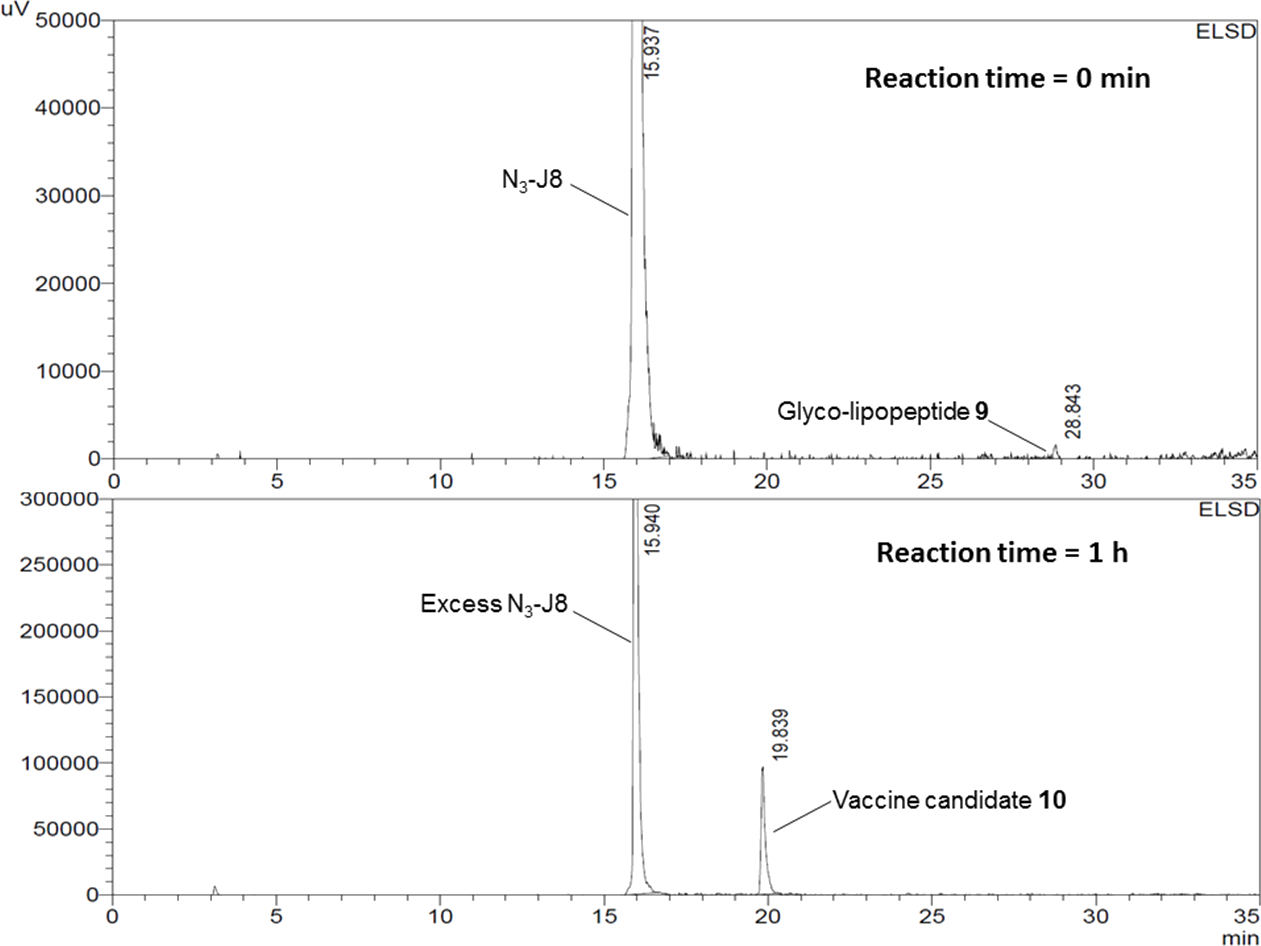
Analytical HPLCs of copper-catalyzed alkyne–azide cycloaddition reaction at the start (top) and after 1 h (bottom) (gradient; 0–100% solvent B over 30 min).

The lipidic adjuvanting moiety was synthesized by standard Fmoc SPPS using the lipidated lysine amino acid **8** (K(C_12_)). This lipidic sequence, lysine lipid core peptide (LLCP; K(C_12_)-G-K(C_12_)-K(C_12_)-G), is enantiomerically pure and analogous to the LCP sequence (C_12_-G-(C_12_)-(C_12_)-G) which has been shown to be an effective adjuvanting moiety [11]. The new carbohydrate carrier **1** was coupled to the LLCP sequence, the intermediate was cleaved from the resin using standard Fmoc SPPS cleavage conditions and the product, glyco-lipopeptide **9**, was used in the final reaction step without purification. Four copies of the N_3_-J8 epitope were then coupled to glyco-lipopeptide **9** using the copper-catalyzed alkyne–azide cycloaddition reaction (Scheme 4), which was performed according to Urbani et al. [35–36]. The progress of the reaction was monitored by analytical RP-HPLC (C4 column, 0–100% solvent B (CH_3_CN/10% H_2_O/0.1% TFA)) and was complete in approximately 1 h (Figure 1). After purification by RP-HPLC vaccine candidate **10** was obtained in 32% yield and characterized by mass spectrometry and RP-HPLC (Supporting Information File 1).

**Scheme 5:**
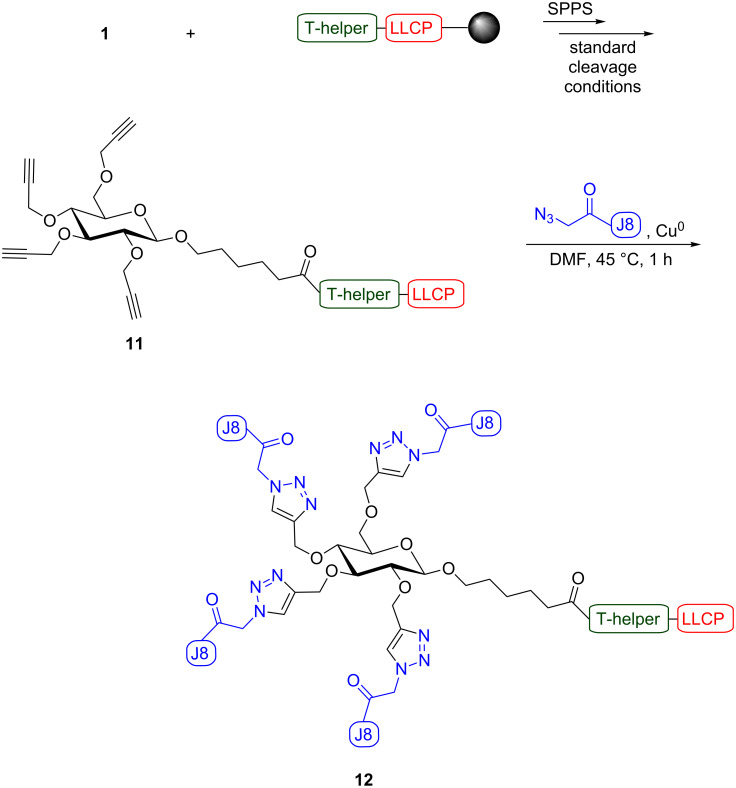
Convergent synthesis of self-adjuvanting vaccine candidate **12** consisting of lipidic adjuvanting moiety LLCP, T-helper epitope, carbohydrate carrier and four copies of J8 B cell epitope.

After the successful synthesis of vaccine candidate **10**, further complexity was introduced into this system. In this case, following the synthesis of LLCP, stepwise SPPS was continued in order to incorporate a T-helper epitope (Pan DR Epitope; PADRE; KFVAAWTLKAA), resulting in a linear peptide containing the T-helper epitope and the LLCP (Scheme 5). The PADRE T-helper epitope has been shown to be recognized by a high percentage of the human population, and is thus referred to as a promiscuous T-helper epitope [21]. Incorporation of this epitope should allow for T cell-dependent activation of B cells, and lead to a stronger and longer lasting immune response. The carbohydrate carrier **1** was coupled to the N-terminus of the linear T-helper-LLCP peptide, the intermediate was cleaved from the resin using standard Fmoc SPPS cleavage conditions and the product, glyco-lipopeptide **11**, was used in the final step without purification (Scheme 5). The copper-catalyzed cycloaddition reaction was performed as described before and gave the desired self-adjuvanting vaccine candidate **12**, the identity and purity of which was confirmed by mass spectrometry and analytical HPLC (Supporting Information File 1).

## Conclusion

A concise and convenient synthesis of a versatile carbohydrate building block was devised. A very efficient procedure for the lipidation of Fmoc-lysine is reported, which may find widespread application for the preparation of lipopeptides. Using these building blocks, a new synthetic methodology was developed, and complex structures were prepared that contain all the necessary components of a self-adjuvanting vaccine in one pure compound. Purification of glyco-lipopeptide **9** and **11** would have significantly improved the yields of the final step, however, this was not required, making the overall synthesis far more efficient. Purification of the final vaccine candidate **10** was achieved in one chromatographic step without difficulty and thus, was less demanding compared to vaccine candidates prepared previously. As a result, the more complex vaccine candidate **12** was synthesized, which contained multiple copies of a B cell epitope coupled to the carbohydrate core, a lipidic adjuvanting moiety, as well as, a promiscuous T-helper epitope to enhance the immune response. Using this synthetic approach a library of GAS vaccine candidates will be synthesized for in vivo evaluation using immunological assays.

## Supporting Information

All experimental procedures are given. ^1^H, ^13^C, DEPT, COSY, HSQC NMR spectra are provided for compounds **4**, **5** and **1**, as well as, analytical RP-HPLC chromatograms and ESIMS spectra for N_3_-J8 peptide, and vaccine candidates **10** and **12**.

File 1 Experimental part.
