# Supplementary material for: Convergent synthetic methodology for the construction of self-adjuvanting lipopeptide vaccines using a novel carbohydrate scaffold
Source: Beilstein J Org Chem. 2014 Jul 30;10:1741–8. doi: 10.3762/bjoc.10.181 (PMC4143089; doi:10.3762/bjoc.10.181)
Supplement: File 1 — Experimental part. [file Beilstein_J_Org_Chem-10-1741-s001.pdf]

Supporting Information

for

Convergent synthetic methodology for the construction of self-adjuvanting lipopeptide vaccines using a novel carbohydrate scaffold

Vincent Fagan[1], Istvan Toth[1,2] and Pavla Simerska*[,1]

Address: [1]The University of Queensland, School of Chemistry and Molecular Biosciences, Cooper Road, St. Lucia QLD 4072, Australia and [2]The University of Queensland, School of Pharmacy, Pharmacy Australia Centre of Excellence, Cornwall Street, Woolloongabba, QLD 4072, Australia

Email: Pavla Simerska* - p.simerska@uq.edu.au

*Corresponding author

Experimental part

Table of Contents	Page
**Experimental** (General)	S3
Synthesis of *Compound* **3**	S5
Synthesis of *Compound* **1**	S5
Characterization of *Compounds* **4**	S6
Characterization of *Compounds* **5**	S7
Synthesis of *Compound* **8**	S7
**Synthesis of Peptides and Lipopeptides** (Standard Fmoc SPPS conditions)	S8
N_3-J8 epitope	S9
Glyco-lipopeptide **9**	S9
Glyco-lipopeptide **11**	S10
**Copper Catalyzed Cycloaddition "Click" Reaction**	S10
Vaccine candidate **10**	S10
Vaccine candidate **12**	S11
NMR *Compound* **4**	S12
NMR *Compound* **5**	S15
NMR *Compound* **1**	S18
Analytical RP-HPLC **N_3-J8**	S21
Mass Spectrum **N_3-J8**	S22
Analytical RP-HPLC Vaccine Candidate **10**	S23
Mass Spectrum Vaccine Candidate **10**	S24
Analytical RP-HPLC Vaccine Candidate **12**	S25
Mass Spectrum Vaccine Candidate **12**	S26
**References**	S27

Experimental

General

Methanol, dimethylformamide (DMF), diethyl ether (Et_2O), acetonitrile, ethyl acetate (EtOAc), hexane, trifluoroacetic acid (TFA), piperidine and *O*-benzotriazol-1-yl)-*N*,*N*,*N′*,*N′*-tetramethyluronium hexafluorophosphate (HBTU) were obtained from Merck KGaA (64271 Darmstadt, Germany). Dichloromethane, dimethyl sulfoxide (DMSO), NaOH and sodium hydrogen carbonate ($NaHCO_3$) were purchased from Chem-Supply (Adelaide, 5013 Australia). Alfa Aesar (Heysham LA3 2XY, England) supplied NaH 57–63% oil dispersion. *O*-(7-Azabenzotriazol-1-yl)-*N*,*N*,*N′*,*N′*-tetramethyluronium hexafluorophosphate (HATU) and all *N*-*α*-Fmoc-protected amino acids were supplied by Mimotopes (Victoria, 3168 Australia). Rink amide 4-methylbenzhydrylamine (MBHA) resin was purchased from Novabiochem (Läufelfingen, Switzerland). Ultra-pure N_2 gas was supplied by BOC Gases (Brisbane, QLD, Australia). Automated flash chromatography was carried out using a Reveleris® X2 flash chromatography system (Grace Materials Technologies, Discovery Sciences) with UV and Evaporative Light Scattering Detection (ELSD). Davisil® Chromatographic Silica Media (LC60A 20–45 micron) was purchased from Grace Materials Technologies, Discovery Sciences (Victoria 3178, Australia) and used for dry column vacuum chromatography (DCVC). Deuterated chloroform ($CDCl_3$) was purchased from Cambridge Isotope Laboratories Inc. (Andover, MA, USA). All other reagents were purchased in analytical grade or higher purity from Sigma-Aldrich (Castle Hill, VIC, Australia). All solvents and reagents were used as purchased unless otherwise stated.

Microwave-assisted peptide couplings were performed using a CEM Discover Bio System manual peptide synthesiser (Matthews, USA). Mass spectra (MS) were obtained from a quadrupole electrospray (Perkin Elmer Sciex API 3000 instrument) in the positive ion mode using Analyst 1.4 (Applied Biosystems/MDS Sciex, Toronto, Canada) software. High

resolution mass spectra (HRMS) were run on an ABSCIEX Triple TOF 5600 Mass Spectrometer. Optical rotations were performed on a JASCO P-2000 polarimeter. Nuclear magnetic resonance (NMR) spectra were recorded on a Bruker AM 300 MHz (1D spectra) or Bruker AM 500 MHz (2D spectra) instrument, using $CDCl_3$ as deuterated solvent with reference to tetramethylsilane as internal standard, at 297 K. Coupling constants are given in Hertz (Hz).

Analytical RP-HPLC was performed on a Shimadzu (Kyoto, Japan) instrument (LC-20AT liquid chromatograph, CMB-20A communication bus module, SIL-20A HT auto sampler, CTO-20 column oven, SPD-M20A diode array detector, ELSD-LT II low temperature evaporative light scattering detector, LabSolutions software), with C18 or C4 Grace Vydac® (Columbia, Maryland, USA) columns (10 µm, 4.6 mm internal diameter × 250 mm,) using a stated gradient of CH_3CN/10% H_2O/0.1% TFA (solvent B) and H_2O/0.1% TFA (solvent A) with a 1 mL/min flow rate and detection at 214 nm.

Semi-preparative RP-HPLC was performed using a Waters Delta 600 system (Milford, Massachusetts, USA; model 600 controller, 490E UV detector). Purifications were achieved using Vydac® C4 or C18 preparative columns (10 µm, 10 mm i.d. × 250 mm) using a stated gradient quoted as % solvent B at 4 mL/min flow rate, and detection at 214 nm. Fractions containing pure compound were pooled and lyophilized overnight.

Preparative RP-HPLC was performed on a Shimadzu (Kyoto, Japan) instrument (LC-20AP liquid chromatograph, CMB-20A communication bus module, SPD-20 UV–vis detector, FRC-10A fraction collector), with C18 or C4 Grace Vydac® (Columbia, Maryland, USA) columns (10 µm, 22 mm internal diameter × 250 mm,) using a stated gradient quoted as % solvent B with a 20 mL/min flow rate and detection at 214 nm.

Methyl 6-hydroxyhexanoate (3):

Concentrated sulfuric acid (1 mL) was added to a solution of ε-caprolactone (10.00 g, 87.61 mmol) in CH_3OH (30 mL) and the mixture was stirred at room temperature for 30 min. The mixture was diluted with Et_2O (100 mL), and the organic phase was washed with sat. $NaHCO_3$ (2 x 50 mL) and H_2O (50 mL). The combined organic extracts were dried ($MgSO_4$) and evaporated to give compound **3** as a colorless oil (10.37 g, 81%); ^1H NMR ($CDCl_3$, 300 MHz) δ 3.63 (s, 3H, OCH_3), 3.60 (t, J = 6.5 Hz, 2H, CH_2-6), 2.29 (t, J = 7.4 Hz, 2H, CH_2-2), 1.67-1.50 (m, 4H, 2 x CH_2), 1.41-1.30 (m, 2H, CH_2); ^{13}C NMR ($CDCl_3$, 75 MHz) δ 172.0 (C=O), 62.5 ($HOCH_2$), 51.5 (OCH_3), 34.0, 32.3, 25.3, 24.6 (all CH_2) [1].

2,3,4,6-Tetra-*O*-propargyl-1-*O*-(6-hydroxy-6-oxohexyl)-β-D-glucopyranose (1):

Tetra-*O*-acetyl-α-D-glucopyranosyl bromide **2** [2], methyl 6-hydroxyhexanoate **3** (1.71 g, 11.69 mmol), CH_2Cl_2 (30 mL) and molecular sieves (4 Å, approx. 4 g) were stirred under N_2 at room temperature overnight. Silver(I) oxide (3.61 g, 15.58 mmol) was added and the mixture was stirred at room temperature for 5 h, which resulted in a mixture of orthoester by-product **4** and the desired glycosylation product **5**. TMSOTf (0.14 mL, 0.79 mmol) was added to the reaction mixture and, after 5 min stirring at room temperature, the solids were removed by filtration through a bed of Celite®. The filtrate was washed with sat. $NaHCO_3$ (2 x 50 mL), dried ($MgSO_4$) and evaporated. The residue was dissolved in CH_3OH (50 mL), Na (0.30 g, 13 mmol) was added and the mixture was stirred under N_2 for 20 min. The mixture was neutralized using Amberlite® IR-120H ion-exchange resin, filtered and the solvent evaporated. Water (50 mL) was added to the residue and the aqueous phase was washed with Et_2O (2 x 50 mL). The aqueous solution was lyophilized and the residue was dissolved in dry THF (50 mL). Sodium hydride (57-63% oil dispersion, 3.12 g, 77.90 mmol) was added and the mixture stirred at room temperature under N_2 for 20 min. Following addition of propargyl

bromide (80% in toluene, 13.02 mL, 116.85 mmol), the reaction mixture was stirred at room temperature overnight. Sodium hydroxide (2 M, 50 mL) was added and the mixture was stirred at room temperature for 1 h. The reaction mixture was transferred to a separating funnel and the basic aqueous phase was washed with Et_2O (3 x 50 mL). The basic phase was acidified with glacial acetic acid and the product was extracted into CH_2Cl_2 (3 x 50 mL). The organic phase was washed with water (50 mL), dried ($MgSO_4$), filtered and evaporated. The residue was purified by automated flash chromatography (0-10% EtOAc in CH_2Cl_2 over 50 mins) to give compound **1** (1.07 g, 31%) as a colorless oil; $[\alpha]^{24,WI}$ +3 (*c* = 0.6, $CHCl_3$); R_f 0.42 (EtOAc/CH_2Cl_2, 1:4); ^{1}H NMR ($CDCl_3$, 300 MHz) δ 4.58-4.34 (m, 6H, 3 x $OC\mathit{H}_2CCH$), 4.29-4.16 (m, 3H, H-1, $OC\mathit{H}_2CCH$), 3.94-3.83 (m, 2H, OC*H*H, C*H*H-6), 3.77 (dd, *J* = 10.7, 4.7 Hz, 1H, CH*H*-6), 3.58-3.29 (m, 5H, H-2, H-3, H-4, H-5, OCH*H*), 2.48-2.44 (m, 4H, 4 x CCH), 2.37 (t, *J* = 7.4 Hz, 2H, $C\mathit{H}_2CO_2CH_3$), 1.72-1.59 (m, 4H, 2 x CH_2), 1.48-1.38 (m, 2H, CH_2); ^{13}C NMR ($CDCl_3$, 75 MHz) δ 179.1 (C=O), 103.0 (C-1), 83.2 (C-3), 81.3 (C-2), 80.0, 79.9, 79.8 (all *C*CH), 79.5 (C*C*H), 77.3 (*C*CH), 76.1 (C-4), 74.8, 74.5, 74.4 (all C*C*H), 74.0 (C-5), 69.7 (OCH_2), 68.4 (C-6), 60.3, 60.1, 59.4, 58.7 (all $\mathit{C}H_2CCH$), 33.8 ($\mathit{C}H_2CO_2CH_3$), 29.2, 25.5, 24.4 (all CH_2); HRMS-ESI (*m/z*): $[M + NH_4]^+$ calcd for $C_{24}H_{34}NO_8$, 464.2284; found 464.229.

Although orthoester byproduct **4** and glycosylation product **5** were not purified, analytical samples were obtained for characterization;

3,4,6-Tri-*O*-acetyl-1,2-*O*-[1-(6-methoxy-6-oxohexyloxy)ethylidene]-α-D-glucopyranose (4); colorless oil; $[\alpha]^{24,WI}$ +26 (*c* = 0.6, $CHCl_3$); R_f 0.42 (EtOAc/hexane, 1:1); ^{1}H NMR ($CDCl_3$, 300 MHz) δ 5.71 (d, *J* = 5.4 Hz, 1H, H-1), 5.19 (t, *J* = 2.9 Hz, 1H, H-3), 4.91 (ddd, *J* = 9.6, 2.7, 0.9 Hz, 1H, H-4), 4.30 (ddd, *J* = 5.4, 3.0, 0.9 Hz, 1H, H-2), 4.21-4.19 (m, 2H, CH_2-6), 3.98-3.91 (m, 1H, H-5), 3.67 (s, 3H, OCH_3), 3.47 (t, *J* = 6.6 Hz, 2H, OCH_2), 2.31 (t,

J = 7.5 Hz, 2H, C$H_2$$CO_2$$CH_3$), 2.12, 2.10, 2.09 (all s, 3H, $COCH_3$), 3.05 (s, 3H, CH_3), 1.69-1.54 (m, 4H, 2 x CH_2), 1.43-1.33 (m, 2H, CH_2); ^{13}C NMR ($CDCl_3$, 75 MHz) δ 174.0, 170.7, 169.7, 169.2 (all C=O), 121.29 (*C*CH_3), 96.9 (C-1), 73.1 (C-2), 70.14 (C-3), 68.2 (C-4), 67.0 (C-5), 66.3 (OCH_2), 63.1 (C-6), 51.5 (OCH_3), 33.9 (*C*$H_2CO_2CH_3$), 29.3, 25.6, 24.6 (all CH_2), 20.8 (4 x CH_3); HRMS-ESI (*m/z*): [M + Na]$^+$ calcd for $C_{21}H_{32}NaO_{12}$, 499.1791; found 499.1780.

2,3,4,6-Tetra-*O*-acetyl-1-*O*-(6-methoxy-6-oxohexyl)-β-D-glucopyranose (5);

Colorless oil; [α]$^{24,\mathrm{WI}}$ -16 (c = 0.3, $CHCl_3$); R_f 0.34 (EtOAc/hexane, 1:1); ^{1}H NMR ($CDCl_3$, 300 MHz) δ 5.20 (t, J = 9.5 Hz, 1H, H-3), 5.08 (t, J = 9.6 Hz, 1H, H-4), 4.98 (dd, J = 9.5, 8.0 Hz, 1H, H-2), 4.48 (d, J = 7.8 Hz, 1H, H-1), 4.26 (dd, J = 12.3, 4.8 Hz, 1H, CH*H*-6), 4.13 (dd, J = 12.3, 2.4 Hz, 1H, C*H*H-6), 3.87 (dt, J = 9.6, 6.2 Hz, 1H, OC*H*H), 3.71-3.66 (m, 4H, H-5, OCH_3), 3.47 (dt, J = 9.6, 6.8 Hz, 1H, OCH*H*), 2.30 (t, J = 7.4 Hz, 2H, C$H_2$$CO_2$$CH_3$), 2.08, 2.04, 2.02, 2.00 (all s, 3H, $COCH_3$), 1.68-1.55 (m, 4H, 2 x CH_2), 1.40-1.32 (m, 2H, CH_2); ^{13}C NMR ($CDCl_3$, 75 MHz) δ 174.0, 170.7, 170.3, 169.4, 169.3 (all C=O), 100.8 (C-1), 72.9 (C-3), 71.8 (C-5), 71.3 (C-2), 69.8 (OCH_2), 68.5 (C-4), 62.0 (C-6), 51.5 (OCH_3), 33.9 (*C*$H_2CO_2CH_3$), 29.1, 25.4, 24.5 (all CH_2), 20.8, 20.64, 20.63, 20.61 (all CO*C*H_3) [3]; HRMS-ESI (*m/z*): [M + Na]$^+$ calcd for $C_{21}H_{32}NaO_{12}$, 499.1791; found 499.1802.

Fmoc-Lysine(lauroyl)-OH (K(C_{12})) (8):

Molecular sieves (4 Å, approx. 4 g) were added to a solution of *para*-toluenesulfonic acid monohydrate (1.36 g, 7.15 mmol) and DIPEA (3.10 mL, 17.80 mmol) in CH_2Cl_2 (30 mL), and the mixture was stirred under N_2 at room temperature for 5 h. Lauroyl chloride (1.40 mL, 6.05 mmol) was added and stirring was continued for 15 min, followed by addition of Fmoc-Lys-OH (2.00 g, 5.43 mmol) and stirring overnight. The solids were removed by filtration through a bed of Celite® and the filtrate was washed with acetic acid (5%, 3 x 50 mL), H_2O

(50 mL x 2), dried ($MgSO_4$) and evaporated. Recrystallization from acetonitrile gave compound **8** (1.96 g, 66%) as a white solid; [α]24,WI +22 (*c* = 1.0, $CHCl_3$); ^{1}H NMR ($CDCl_3$, 300 MHz) δ 7.75 (d, *J* = 7.5 Hz, 2H, 2 x Ar-H, 7.62-7.58 (m, 2H, 2 x Ar-H), 7.34 (t, *J* = 7.4, 2H, 2 x Ar-H), 7.31 (d, *J* = 7.2, 2H, 2 x Ar-H), 5.80-5.75 (m, 2H), 4.40-4.32 (m, 2H), 4.21 (t, *J* = 7.2 Hz, 1H), 3.31-3.22 (m, 2H), 2.17 (t, *J* = 7.5, 2H), 1.98-1.71 (m, 2H), 1.64-1.50 (m, 4H), 1.46-1.40 (m, 2H), 1.29-1.18 (m, 16H), 0.88 (t, *J* = 6.6 Hz, 3H); ^{13}C NMR ($CDCl_3$, 75 MHz) δ 174.8, 174.4, 156.3 (all C=O), 143.7, 141.3 (2 x Ar-C), 127.7, 127.1, 125.1, 119.9 (all Ar-CH), 67.1 (CH_2), 53.6 (CH), 47.1 (CH), 39.1, 36.8, 31.9, 29.7, 29.6, 29.5, 29.4, 29.33, 29.29, 29.0, 25.8, 22.7, 22.2 (all CH_2), 14.1 (CH_3) [4]; HRMS-ESI (*m/z*): $[M + H]^+$ calcd for $C_{33}H_{47}N_2O_5$, 551.3485; found 551.3475.

Synthesis of Peptides and Lipopeptides

Stepwise SPPS (manual and microwave-assisted) was performed using rink amide MBHA resin (substitution ratio: 0.60 mmol/g) with standard Fmoc SPPS chemistry:

Fmoc-amino acids (4.2 equiv.) were pre-activated with HATU (0.4 M in DMF, 4 equiv.), and DIPEA (6.2 equiv.). Coupling cycles consisted of Fmoc deprotection with piperidine (20% in DMF, 2 x 15 min (manual) or 2 x 2 min, 70 ºC (microwave)), DMF washing, followed by coupling with pre-activated Fmoc-amino acids (2 x 30 min (manual) or 2 x 5 min, 70 ºC (microwave)). After assembly of the required sequence, the peptidyl-resin was washed with DMF, CH_2Cl_2 and dried under vacuum. Peptides were cleaved from the resin by stirring in a solution of TFA, triisopropylsilane (TIPS) and H_2O (95:2.5:2.5) for 3 h. The resin was removed by filtration and the excess TFA was evaporated by a stream of N_2. The peptides were precipitated from ice-cold Et_2O, centrifuged and the Et_2O decanted.

N_3-J8 epitope:

The J8 B cell epitope (QAEDKVKQSREAKKQVEKALKQLEDKVQ) was synthesized using the above manual standard Fmoc SPPS conditions. Azidoacetic acid [5] was coupled in triplicate to the N-terminus of the J8 B cell epitope using the same conditions as those used for Fmoc-amino acid couplings. Following cleavage from the resin and precipitation, the crude N_3-J8 epitope was dissolved in distilled H_2O, lyophilized and purified by preparative RP-HPLC on a C18 column with a gradient of 0-25 % over 10 min, 25-30% solvent B over 30 min (0.633 g, 47% purified yield).

HPLC analysis (C18 column): t_R = 28.39 min (0% for 5 min, 0-20% over 5 min, 20-50% solvent B over 30 min); MS-ESI (*m/z*): $[M + 2H]^{2+}$ calcd 1683.4, found 1683.9; $[M + 3H]^{3+}$ calcd 1122.6, found 1122.8; $[M + 4H]^{4+}$ calcd 842.2, found 842.6; $[M + 5H]^{5+}$ calcd 674.0, found 674.2; MW 3364.8 g/mol.

Glyco-lipopeptide (9):

The LLCP lipopeptide (K(C12)GK(C12)K(C12)G) was synthesized using microwave-assisted standard Fmoc SPPS conditions. Carbohydrate carrier **1** (2.5 equiv.) was pre-activated with HBTU (2.4 equiv.) and DIPEA (4.0 equiv.), and coupled to the LLCP lipopeptide with microwave assistance (2 x 10 min at 70 ºC), to give glyco-lipopeptide **9**. Following cleavage from the resin and precipitation, the crude glyco-lipopeptide **9** was dissolved in a minimum volume of DMF, diluted with solvent A and B (1:1, 100 mL), and lyophilized. Glyco-lipopeptide **9** was used without purification in the proceeding step. HPLC analysis (C4 column): t_R = 25.31 (0-50% over 5 min, 50-100% solvent B over 30 min); MS-ESI (*m/z*): $[M + H]^{+}$ calcd 1491.0, found 1491.6; $[M + 2H]^{2+}$calcd 746.1, found 746.5; MW 1490.0 g/mol.

Glyco-lipopeptide (11):

The T-helper-LLCP lipopeptide (KFVAAWTLKAA-K(C12)GK(C12)K(C12)G) was synthesized using microwave-assisted standard Fmoc SPPS conditions. Carbohydrate carrier **1** was coupled as described above, to give glyco-lipopeptide **11**. Following cleavage from the resin and precipitation, the crude glyco-lipopeptide **11** was dissolved in a minimum volume of DMF, diluted with solvent A and B (1:1, 100 mL), and lyophilized. Glyco-lipopeptide **11** was used without purification in the proceeding step. HPLC analysis (C4 column): t_R = 33.50 (0-50% over 10 min, 50-80% solvent B over 30 min); MS-ESI (*m/z*), $[M + H]^+$ *mz* calcd 2679.5, found 2679.9; $[M + 2H]^{2+}$ calcd 1340.3, found 1340.6), $[M + 3H]^{3+}$ calcd 893.8, found 894.3; MW 2678.5 g/mol.

Copper Catalyzed Cycloaddition "Click" Reaction

Vaccine candidate (10):

Copper wire (0.100 g) was added to a solution of N_3-J8 (44.0 mg, 13.0 μmol) epitope and crude glyco-lipopeptide **9** (2.0 mg, 1.3 μmol) in DMF (3 mL), and the mixture was stirred at 45 ºC for 1 h. The copper wire was removed by filtration and the DMF filtrate was loaded directly onto a preparative C4 column (RP-HPLC). The product **10** was eluted with a gradient of 0% for 25 min, 0-20% over 5 min, 20-60% solvent B over 60 min (4.1 mg, 32% purified yield).

HPLC analysis (C4 column): t_R = 28.59 (0% for 5 min, 0-20% over 5 min, 20-60% solvent B over 30 min); MS-ESI (*m/z*): $[M + 5H]^{5+}$ calcd 2991.0, found 2990.2; $[M + 6H]^{6+}$ calcd 2492.7, found 2494.2; $[M + 7H]^{7+}$ calcd 2136.7, found 2137.9; $[M + 8H]^{8+}$ calcd 1869.8, 1870.5; $[M + 9H]^{9+}$ calcd 1662.1, found 1663.2; $[M + 10H]^{10+}$ calcd 1496.0, found 1497.3; $[M + 11H]^{11+}$ calcd 1360.1, found 1360.6; $[M + 12H]^{12+}$ calcd 1246.9, found 1247.7; $[M + 13H]^{13+}$ calcd 1151.0, found 1151.5; MW 14950.2 g/mol.

Vaccine candidate (12):

Copper wire (0.100 g) was added to a solution of N_3-J8 (30.3 mg, 9.0 µmol) epitope and crude glyco-lipopeptide **11** (2.4 mg, 0.9 µmol) in DMF (3 mL), and the mixture was stirred at 45 ºC for 1 h. The copper wire was removed by filtration and the DMF filtrate was loaded directly onto a preparative C4 column (RP-HPLC). The product **12** was eluted with a gradient of 0% for 25 min, 0-20% over 5 min, 20-60% solvent B over 60 min (3.5 mg, 24% purified yield).

HPLC analysis (C4 column): t_R = 31.85 (0% for 5 min, 0-20% over 5 min, 20-60% solvent B over 30 min); MS-ESI (*m/z*): $[M + 9H]^{9+}$ calcd 1794.1, found 1794.9; $[M + 10H]^{10+}$ calcd 1614.8, found 1614.9; $[M + 11H]^{11+}$ calcd 1468.1, found 1468.5; $[M + 12H]^{12+}$ calcd 1345.8, found 1346.3; $[M + 13H]^{13+}$ calcd 1242.4, found 1243.1; $[M + 14H]^{14+}$ calcd 1153.7, found 1154.2; $[M + 15H]^{15+}$ calcd 1076.8, found 1077.3; $[M + 16H]^{16+}$ calcd 1009.6, found 1010.2; $[M + 17H]^{17+}$ calcd 950.3, found 950.5; MW 16137.7 g/mol.

3,4,6-Tri-*O*-acetyl-1,2-*O*-[1-(6-methoxy-6-oxohexyloxy)ethylidene]-α-D-glucopyranose (4)

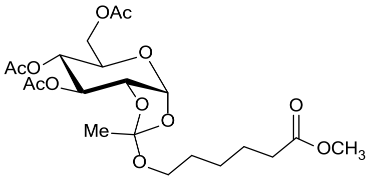

4

^{1}H NMR ($CDCl_3$, 300 MHz)

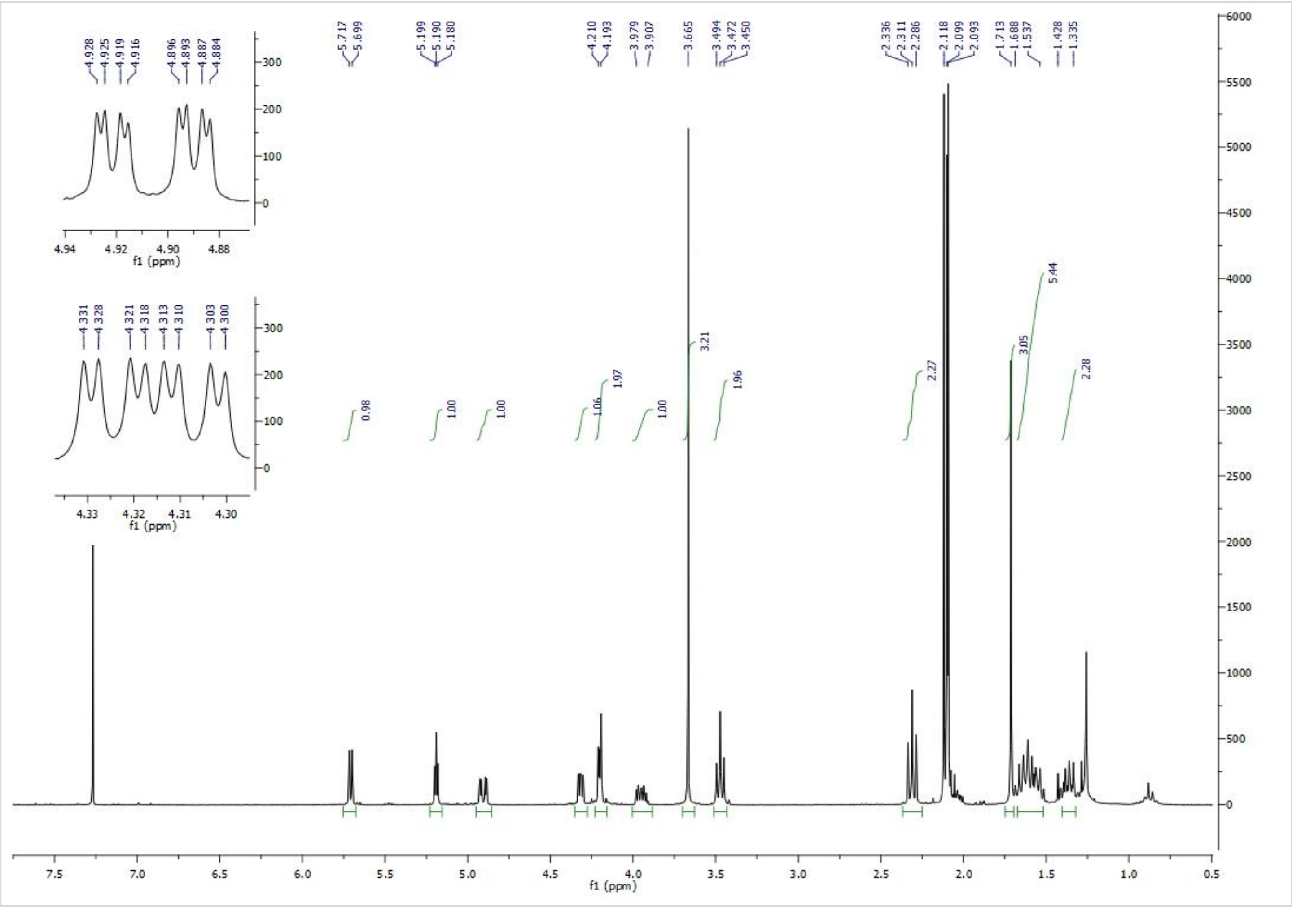

3,4,6-Tri-*O*-acetyl-1,2-*O*-[1-(6-methoxy-6-oxohexyloxy)ethylidene]-α-D-glucopyranose (4)

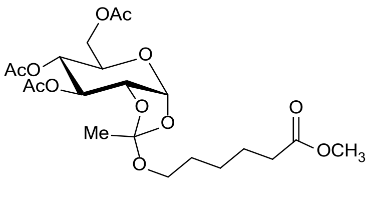

4

^{13}C and DEPT NMR ($CDCl_3$, 300 MHz)

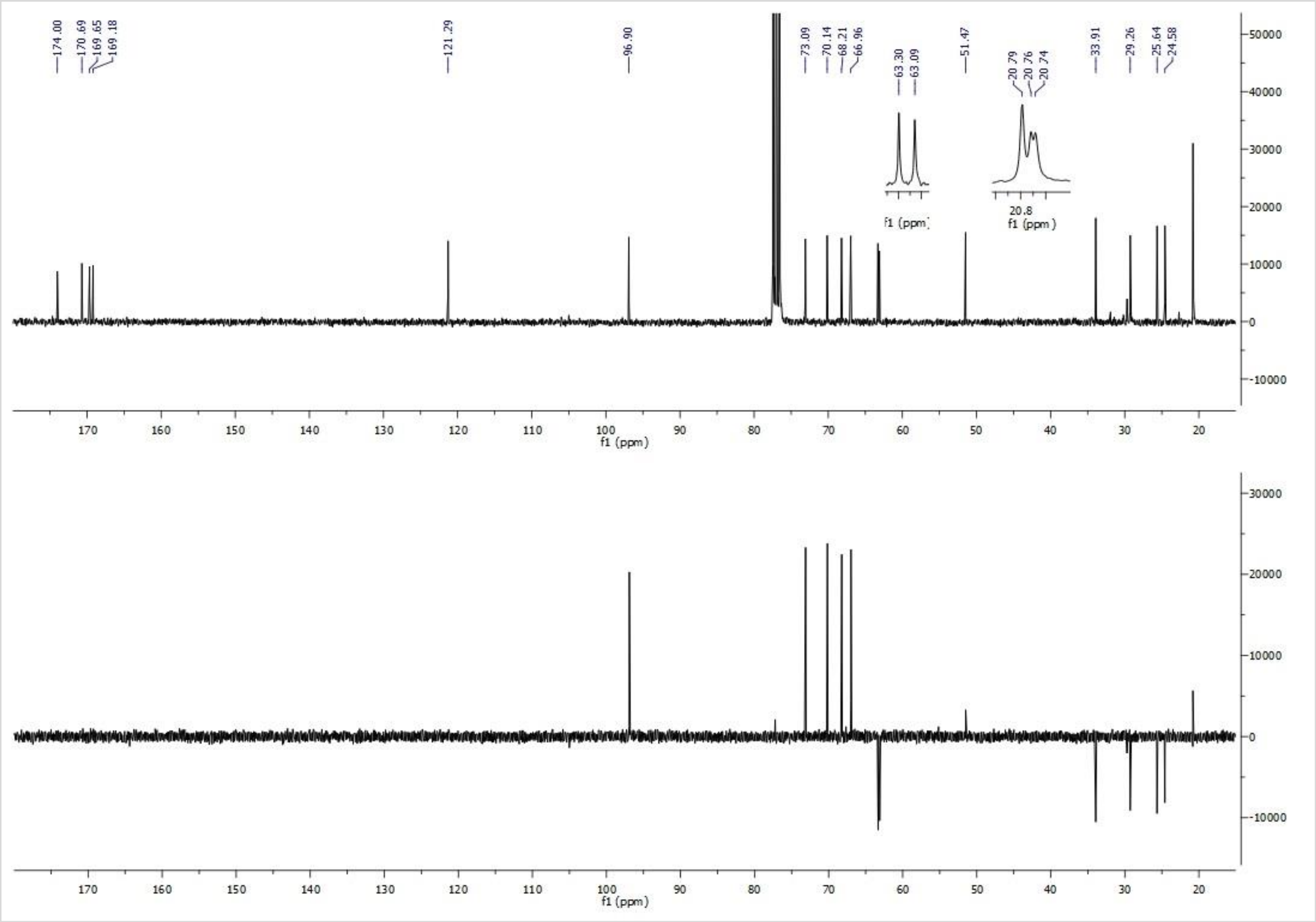

3,4,6-Tri-*O*-acetyl-1,2-*O*-[1-(6-methoxy-6-oxohexyloxy)ethylidene]-α-D-glucopyranose (4)

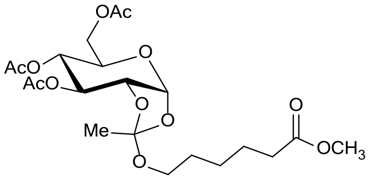

4

COSY and HSQC 2D NMR ($CDCl_3$, 500 MHz)

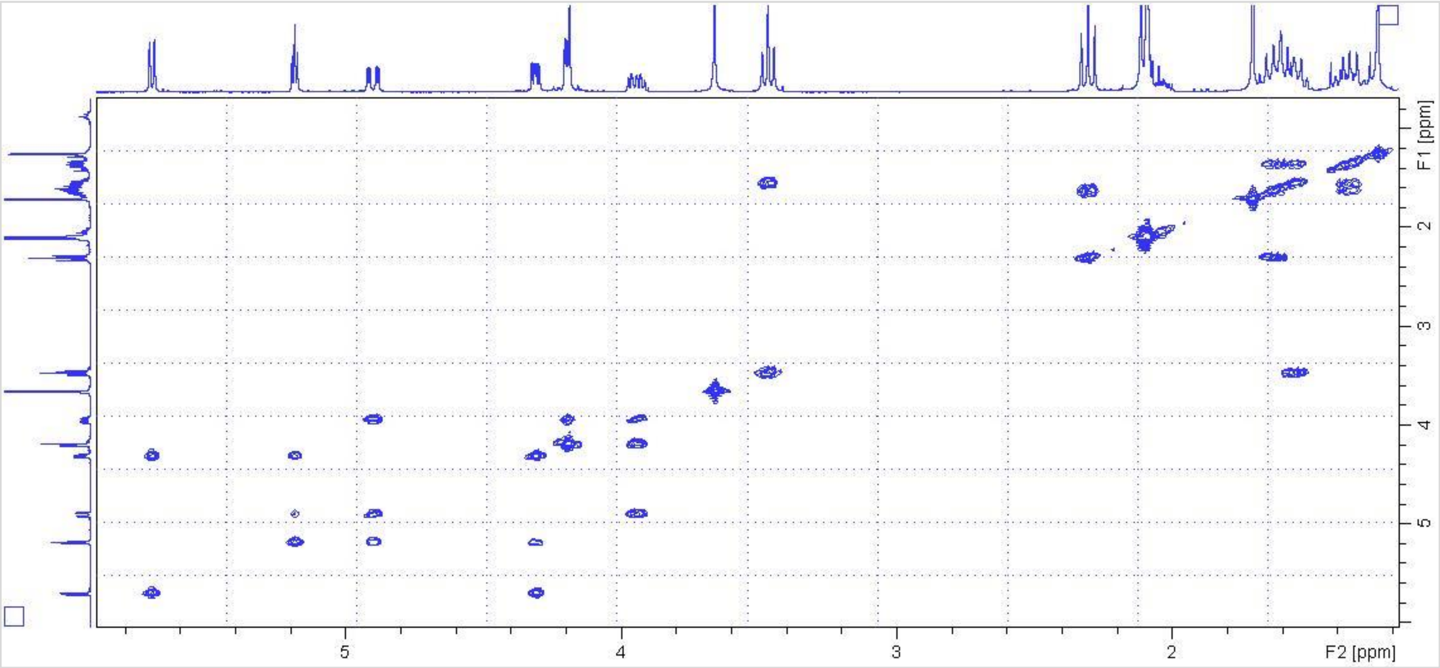

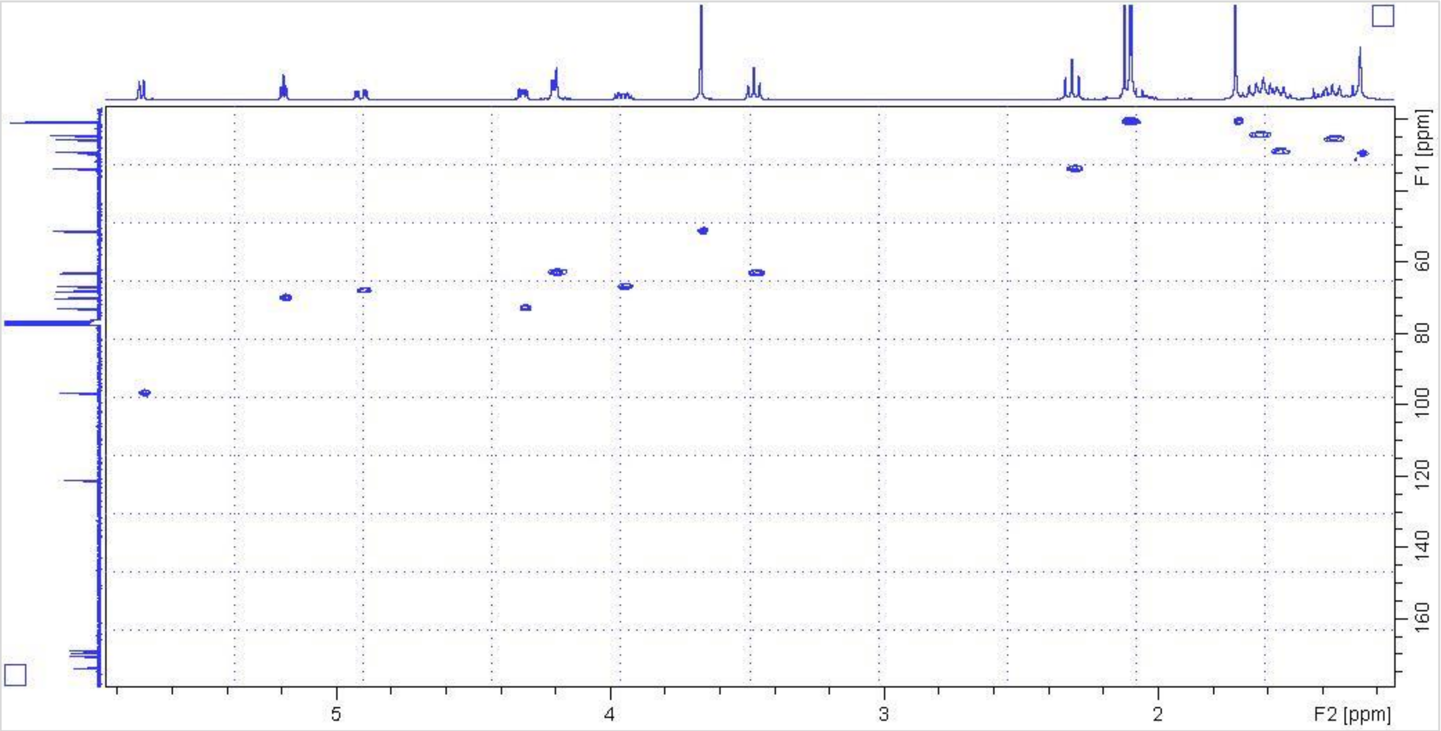

2,3,4,6-Tetra-*O*-acetyl-1-*O*-(6-methoxy-6-oxohexyl)-β-D-glucopyranose (5)

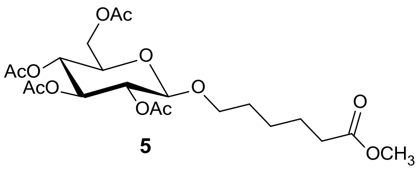

^{1}H NMR ($CDCl_3$, 300 MHz)

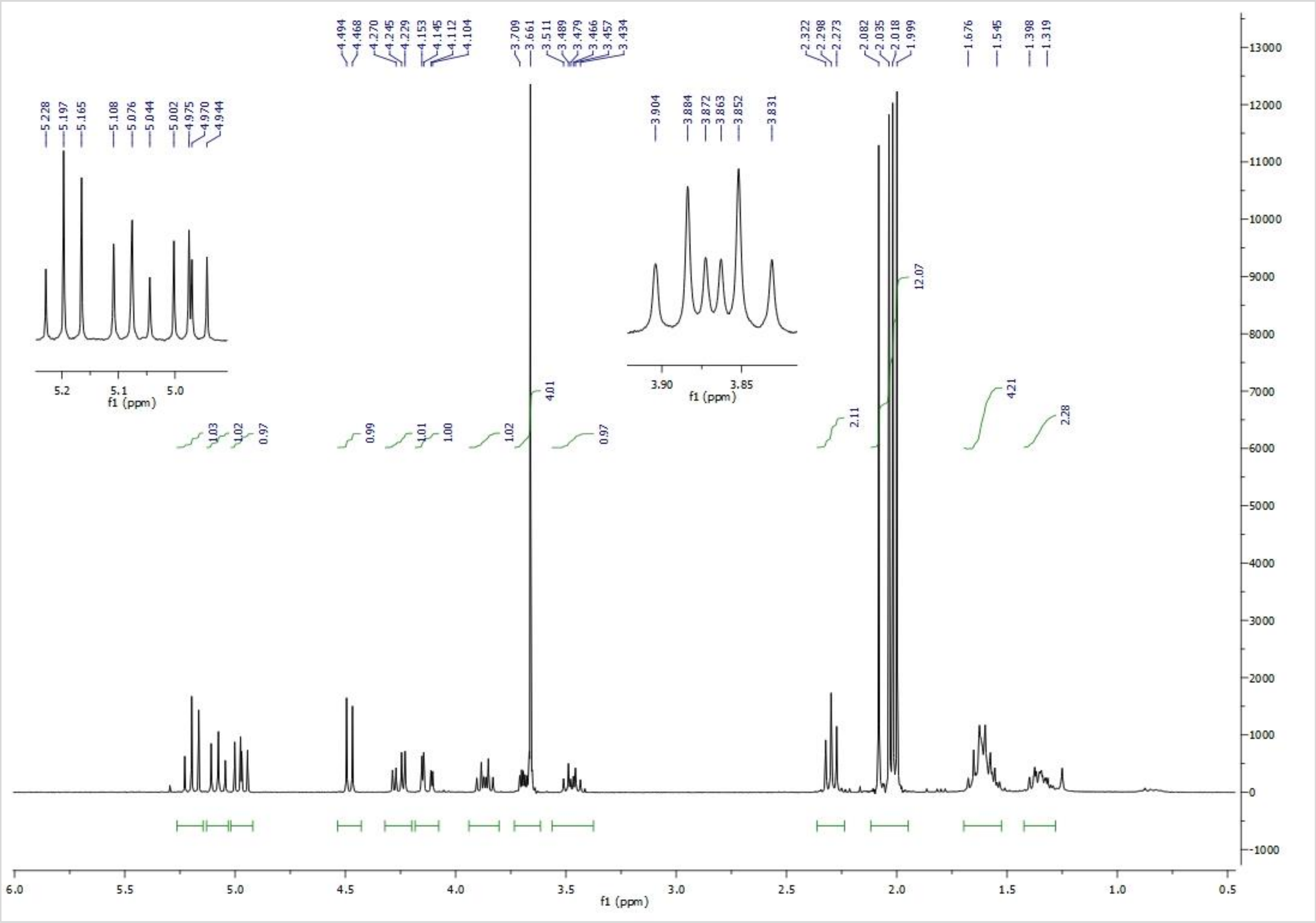

2,3,4,6-Tetra-*O*-acetyl-1-*O*-(6-methoxy-6-oxohexyl)-β-D-glucopyranose (5)

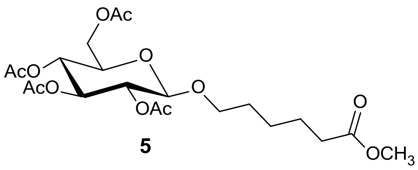

^{13}C and DEPT NMR ($CDCl_3$, 300 MHz)

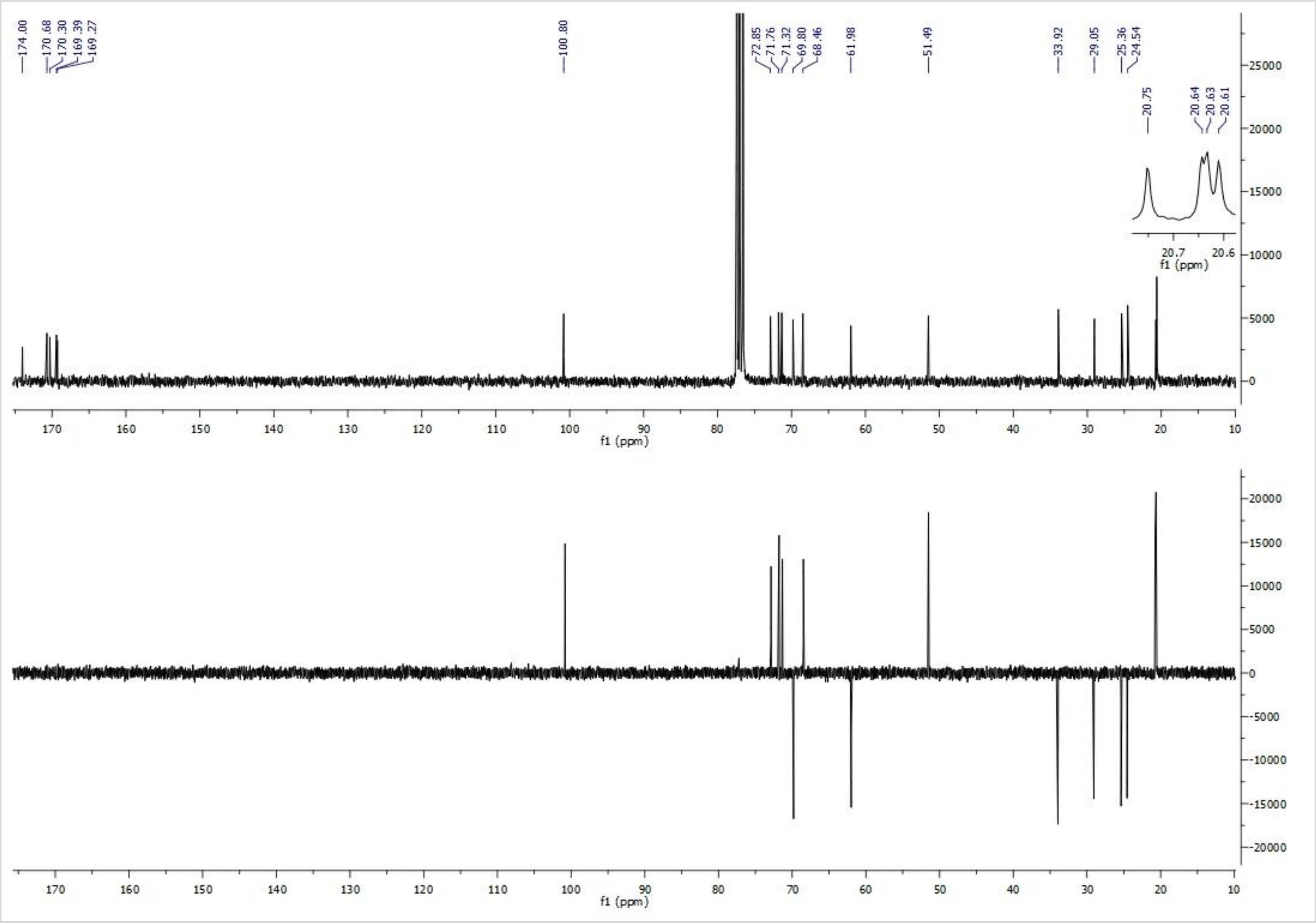

2,3,4,6-Tetra-*O*-acetyl-1-*O*-(6-methoxy-6-oxohexyl)-β-D-glucopyranose (5)

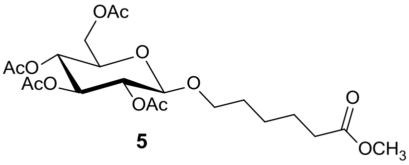

5

COSY and HSQC 2D NMR ($CDCl_3$, 500 MHz)

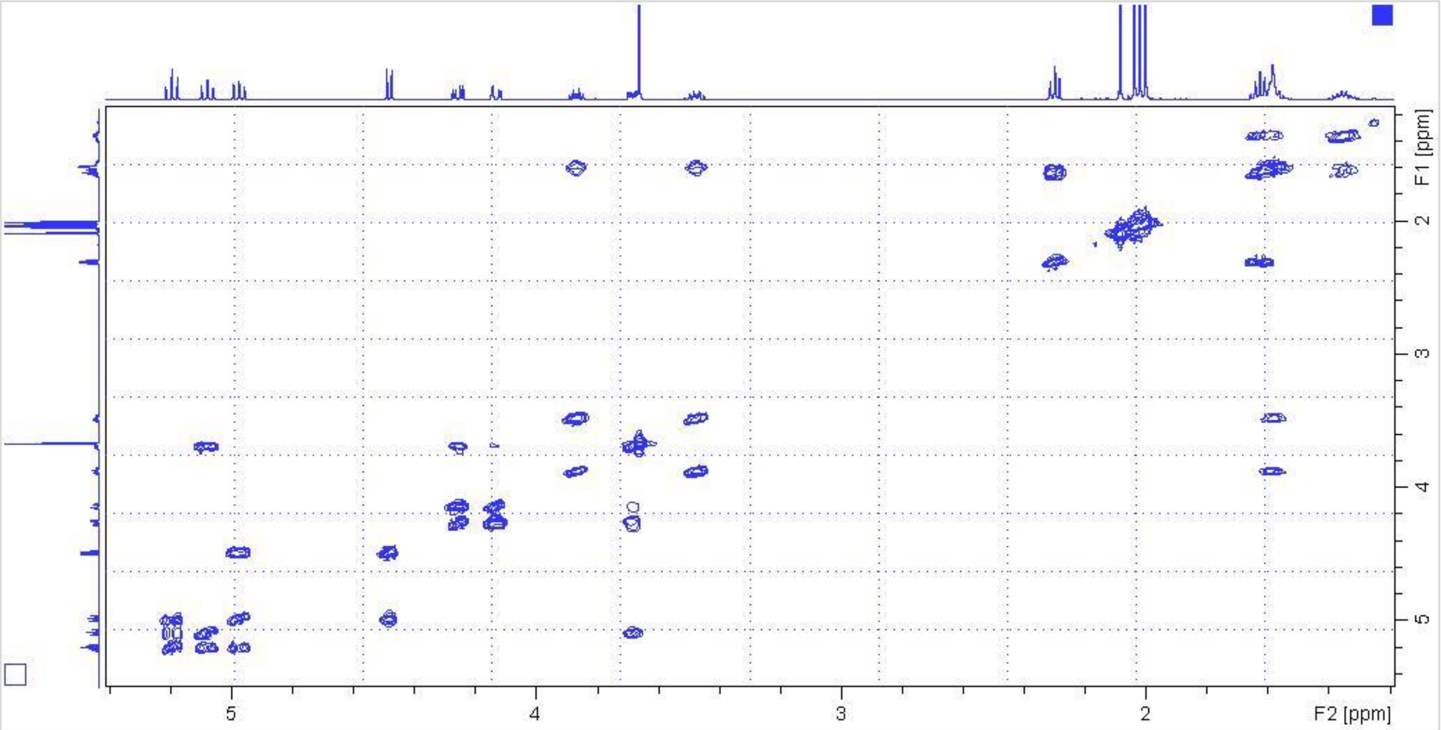

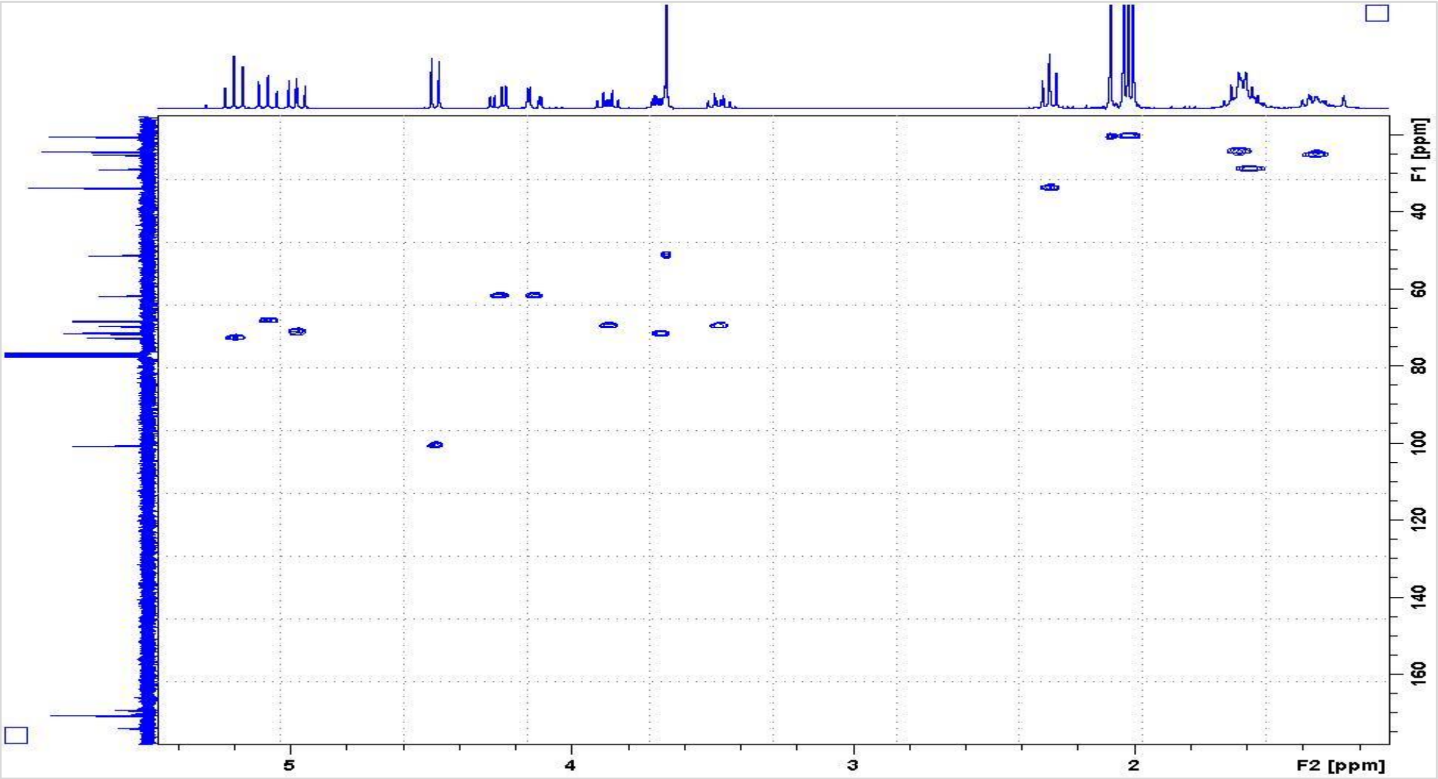

2,3,4,6-Tetra-*O*-propargyl-1-*O*-(6-methoxy-6-oxohexyl)-β-D-glucopyranose (1)

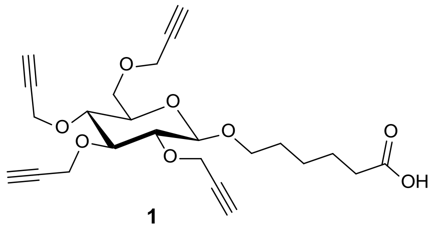

1

^{1}H NMR ($CDCl_3$, 300 MHz)

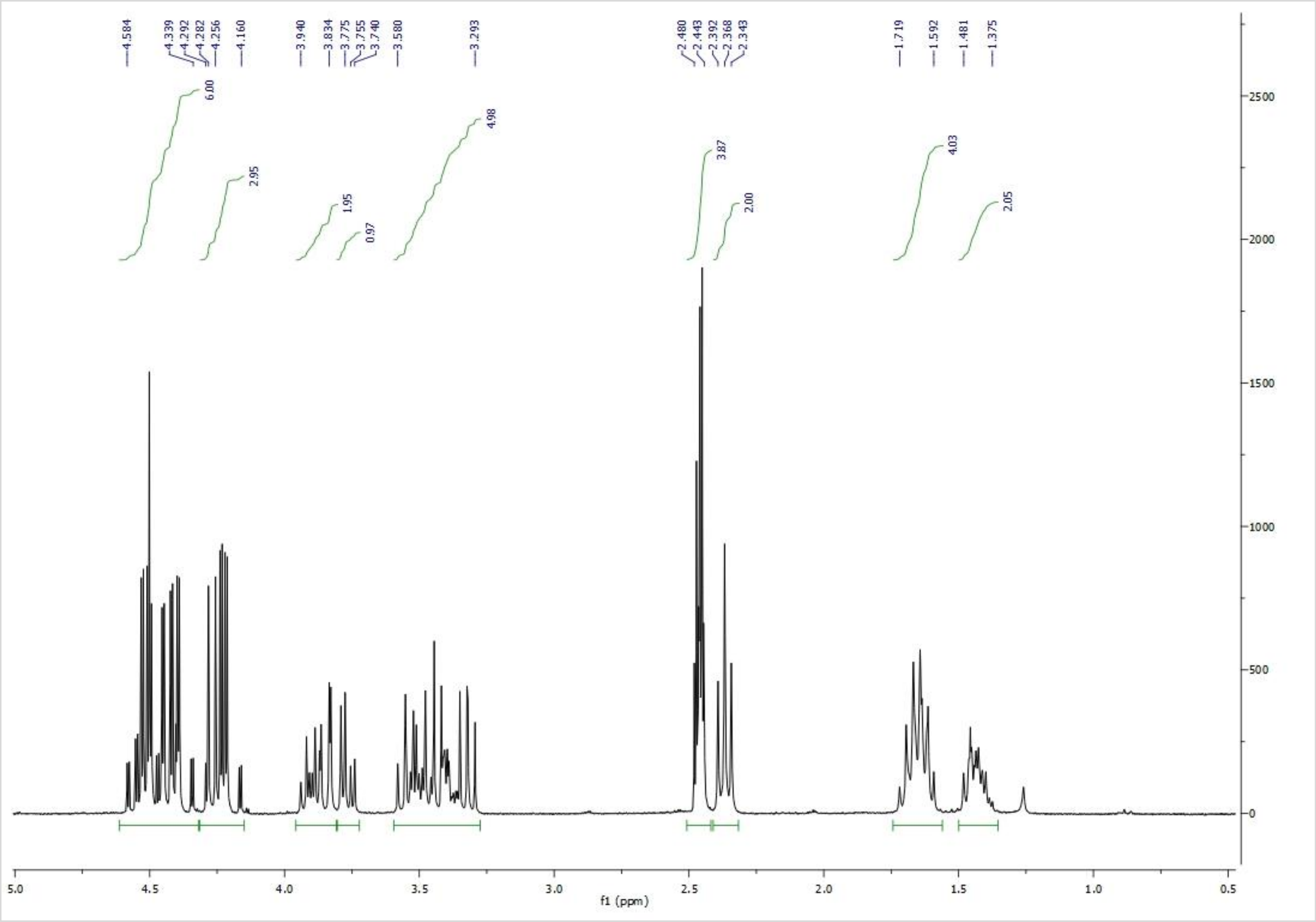

2,3,4,6-Tetra-*O*-propargyl-1-*O*-(6-methoxy-6-oxohexyl)-β-D-glucopyranose (1)

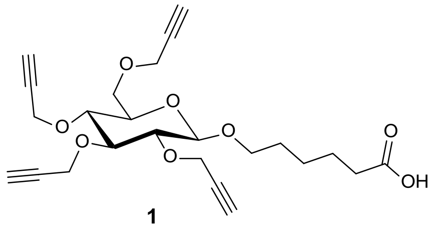

1

^{13}C and DEPT NMR ($CDCl_3$, 300 MHz)

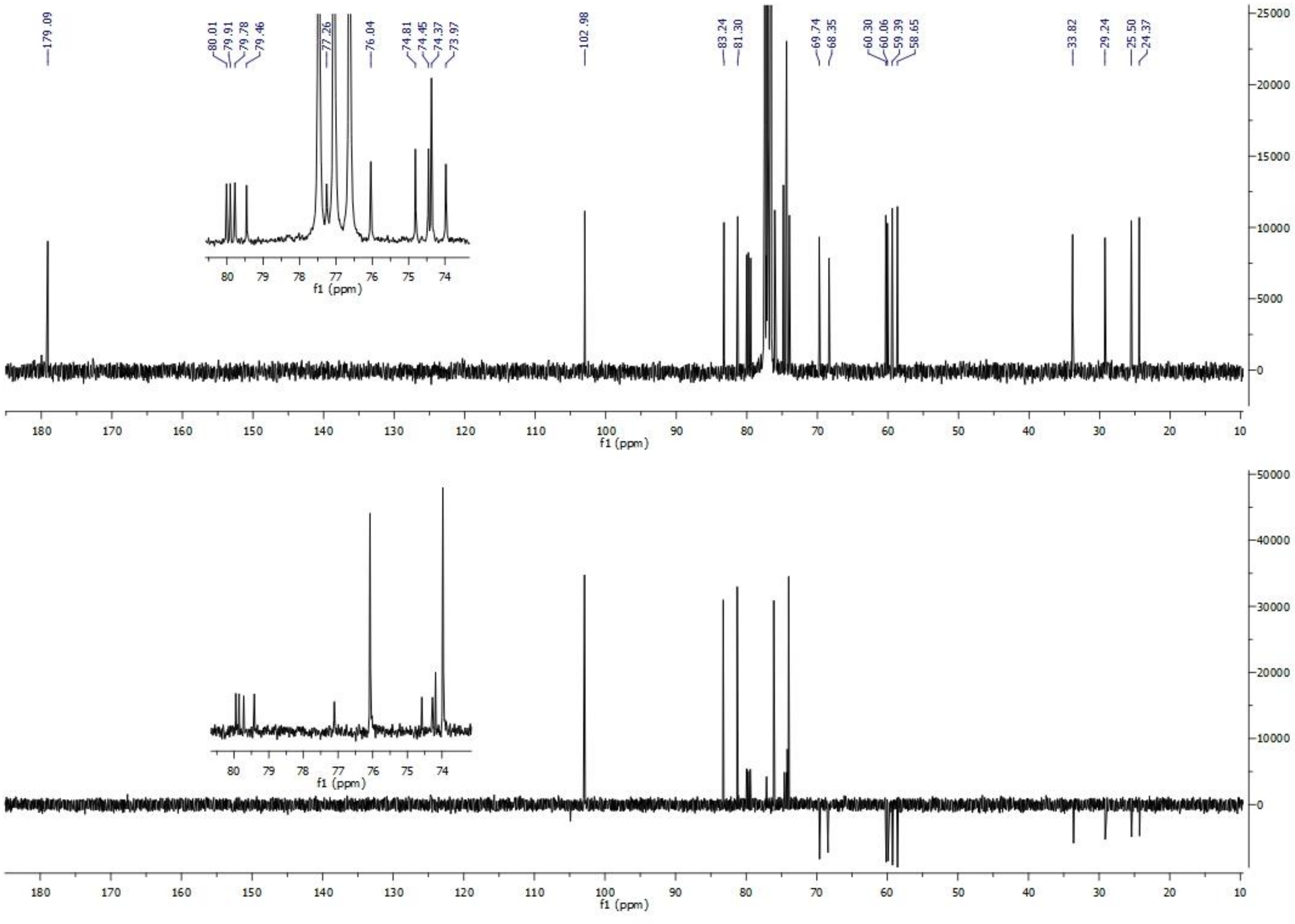

2,3,4,6-Tetra-*O*-propargyl-1-*O*-(6-methoxy-6-oxohexyl)-β-D-glucopyranose (1)

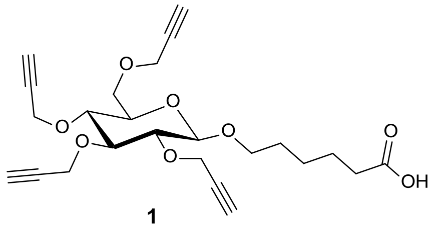

1

COSY and HSQC 2D NMR ($CDCl_3$, 500 MHz)

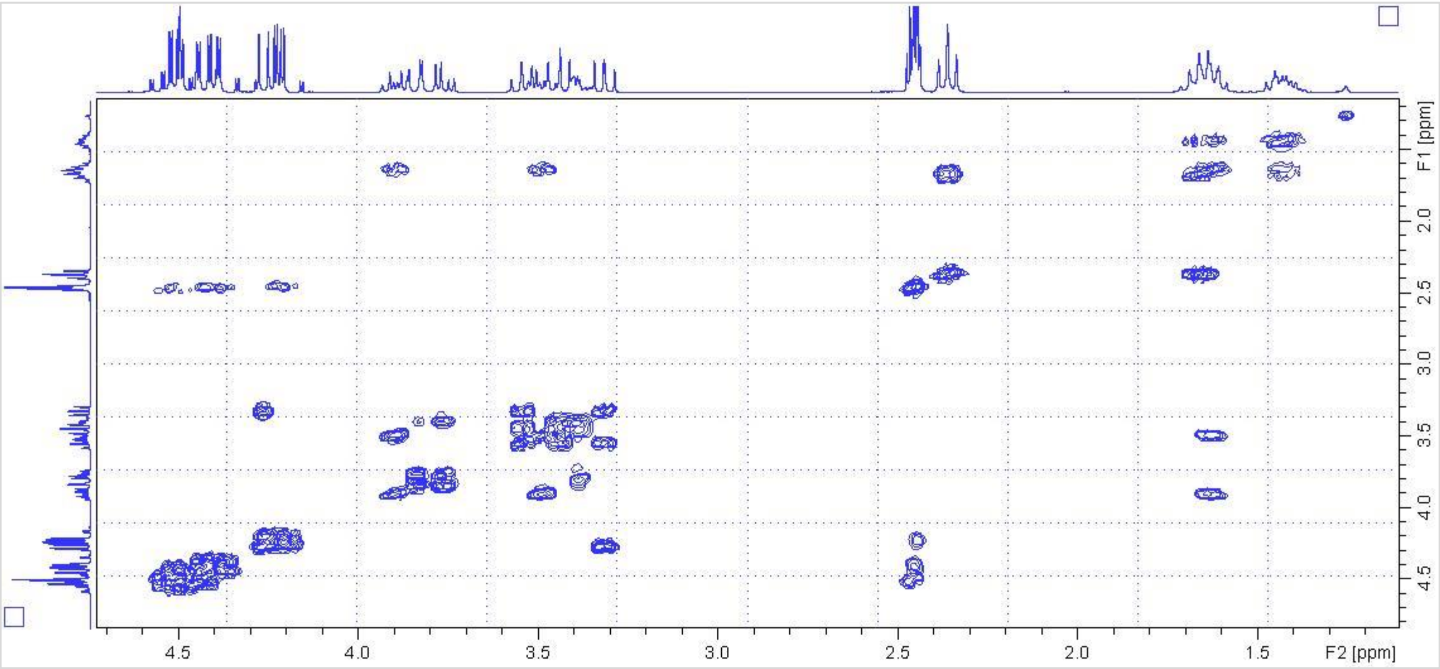

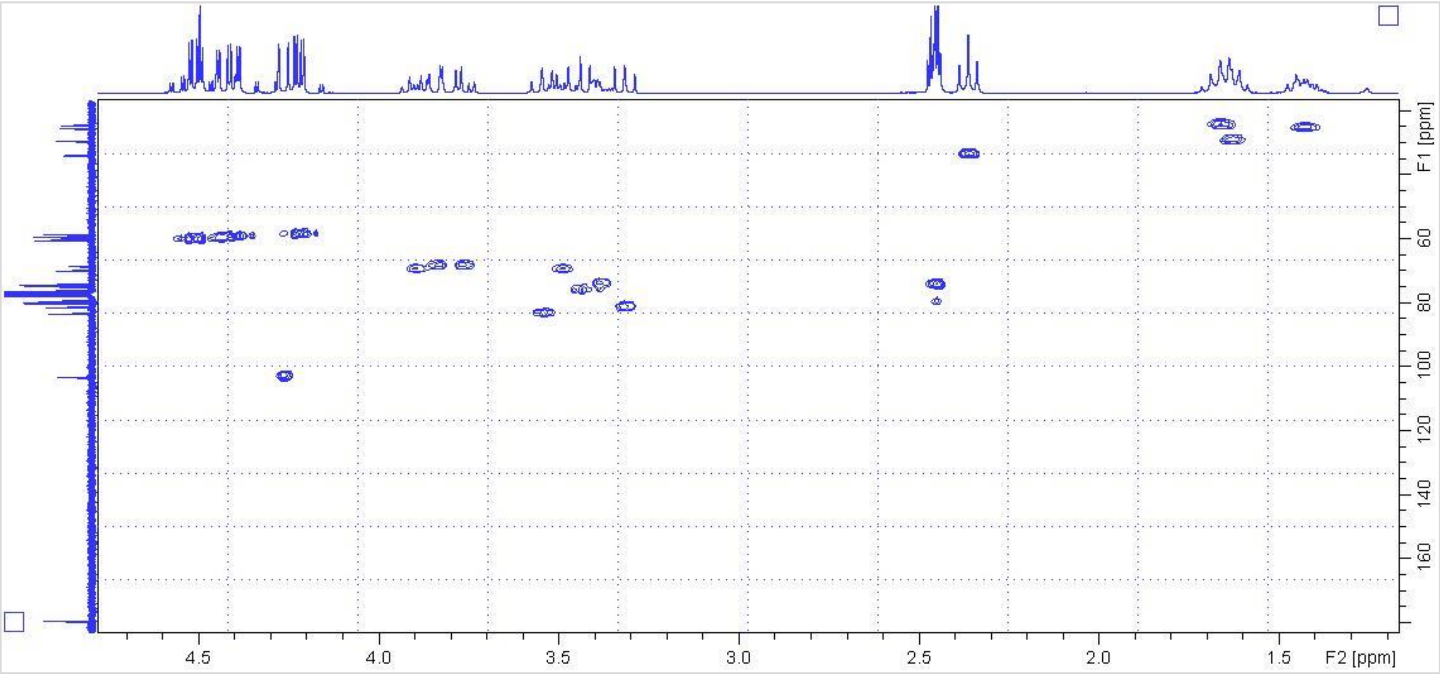

Analytical RP-HPLC chromatograms of N_3-J8 epitope

Blue trace = % solvent B; 5 min at 0%, 0-20% over 5 min, 20-50% over 30 min.

t_R = 28.39 min

UV detection (λ = 214 nm)

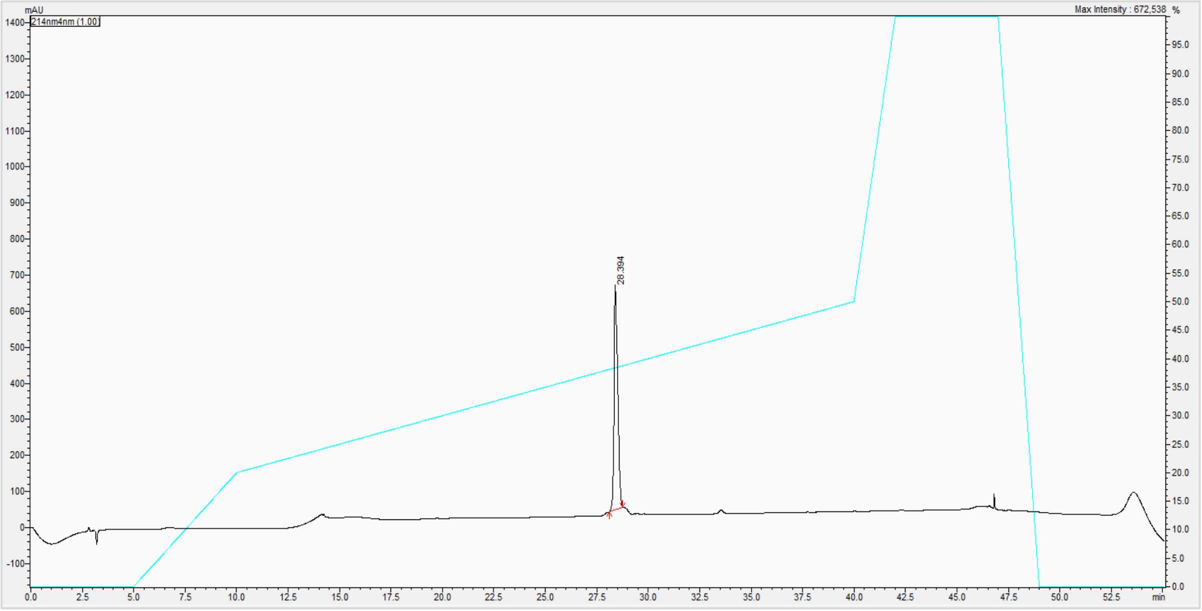

ELSD detection

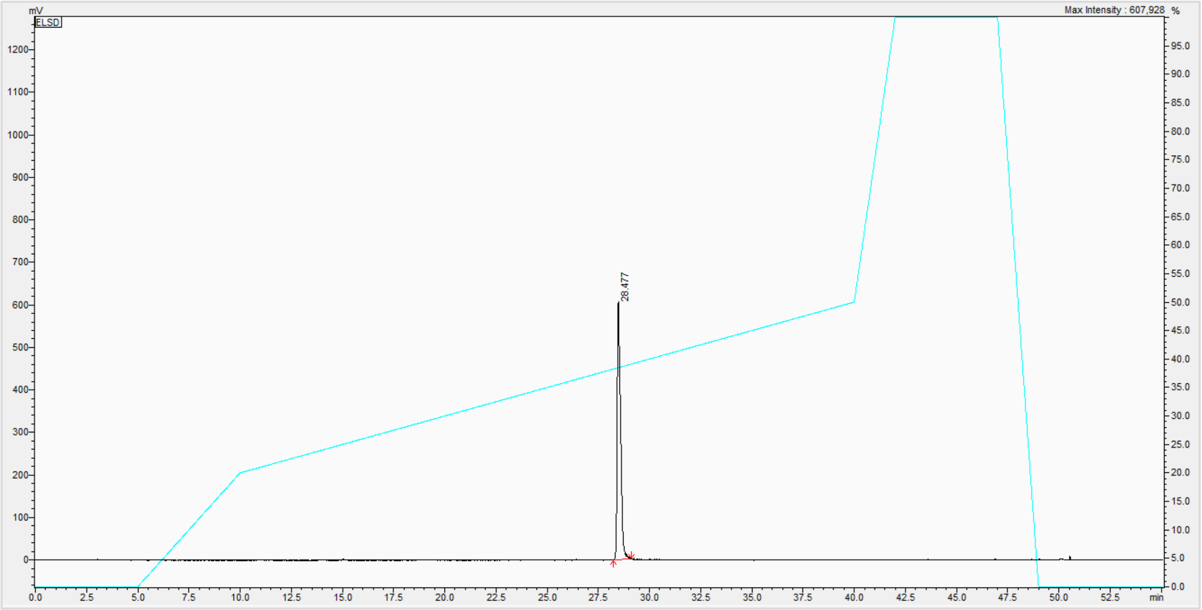

Mass Spectrum of N_3-J8 epitope

Molecular Weight: 3364.8

MS-ESI (*m/z*): $[M + 2H]^{2+}$ calcd 1683.4, found 1683.9; $[M + 3H]^{3+}$ calcd 1122.6, found 1122.8; $[M + 4H]^{4+}$ calcd 842.2, found 842.6; $[M + 5H]^{5+}$ calcd 674.0, found 674.2.

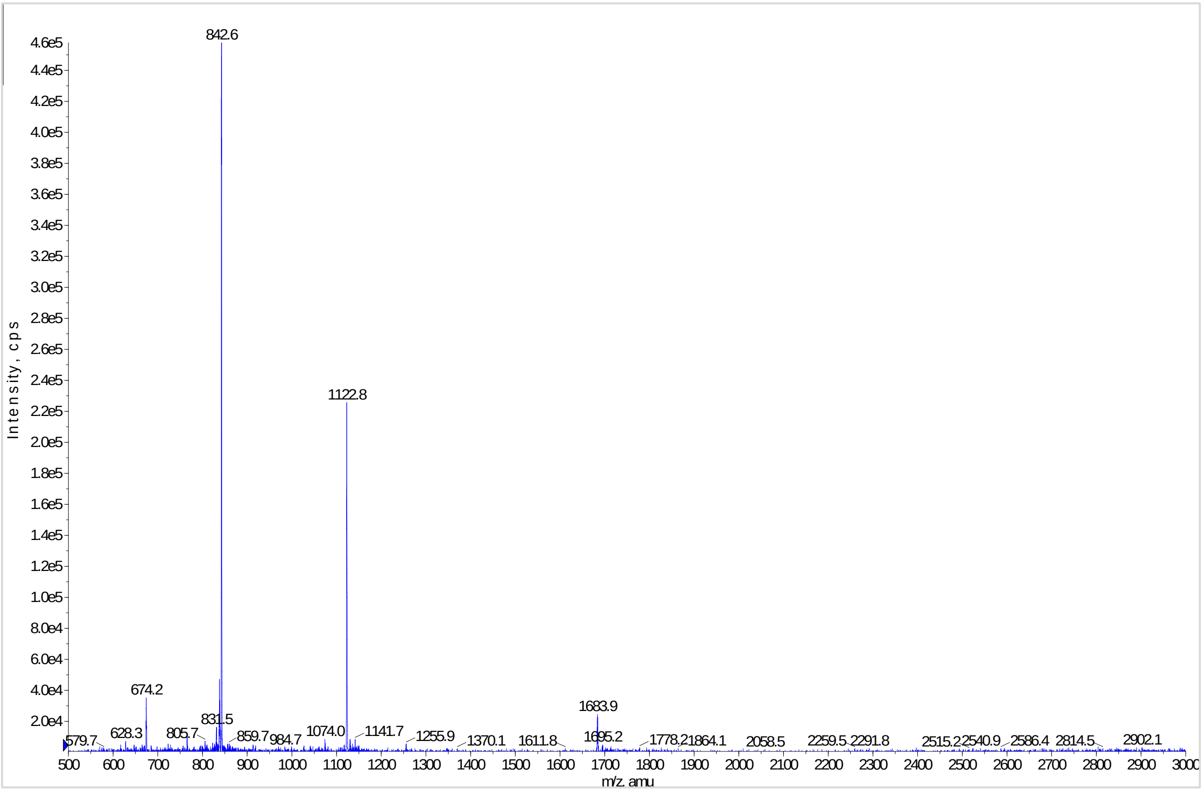

Analytical RP-HPLC chromatograms of Vaccine Candidate 10

Blue trace = % solvent B; 5 min at 0%, 0-20% over 5 min, 20-60% over 30 min.

t_R = 28.59 min

UV detection (λ = 214 nm)

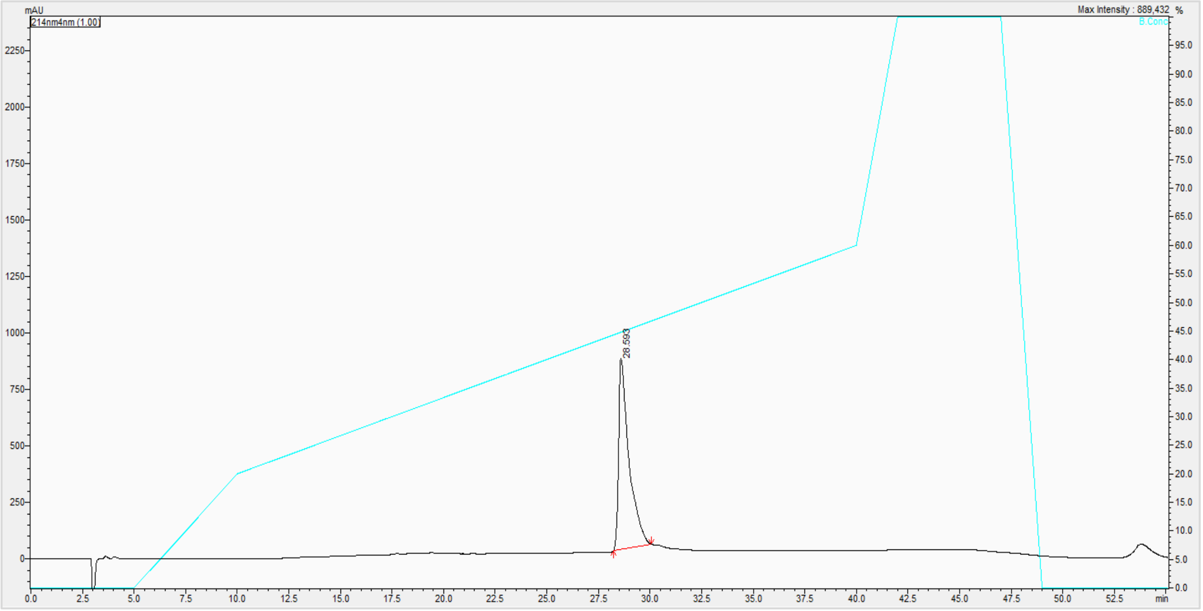

ELSD detection

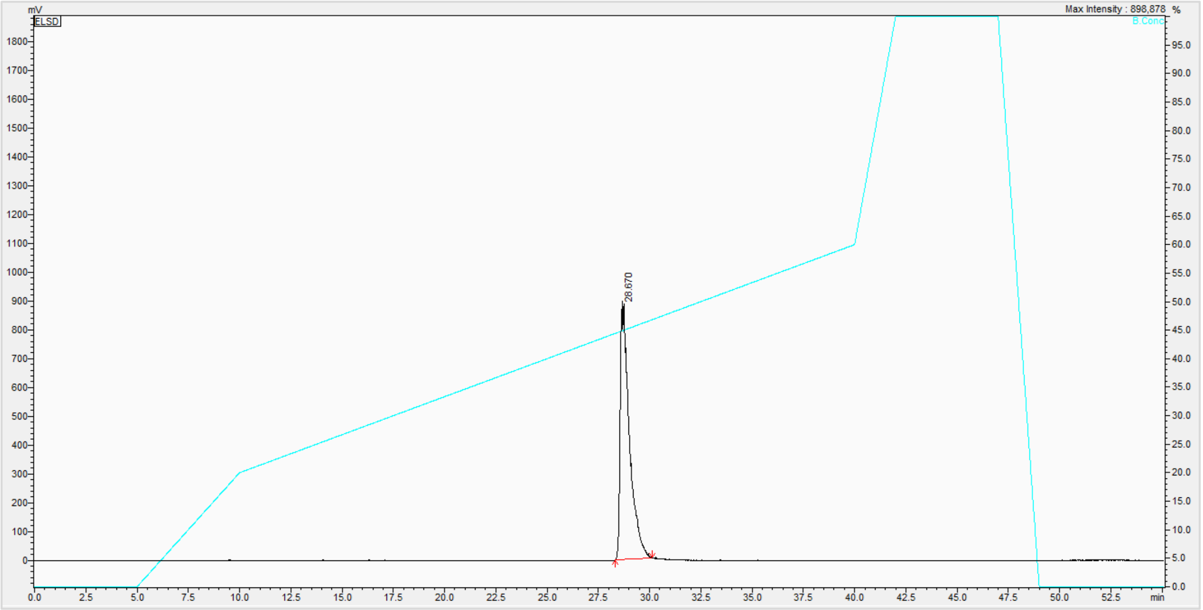

Mass Spectrum of Vaccine Candidate 10

Molecular Weight: 14950.2

MS-ESI (*m/z*): $[M + 5H]^{5+}$ calcd 2991.0, found 2990.2; $[M + 6H]^{6+}$ calcd 2492.7, found 2494.2; $[M + 7H]^{7+}$ calcd 2136.7, found 2137.9; $[M + 8H]^{8+}$ calcd 1869.8, 1870.5; $[M + 9H]^{9+}$ calcd 1662.1, found 1663.2; $[M + 10H]^{10+}$ calcd 1496.0, found 1497.3; $[M + 11H]^{11+}$ calcd 1360.1, found 1360.6; $[M + 12H]^{12+}$ calcd 1246.9, found 1247.7; $[M + 13H]^{13+}$ calcd 1151.0, found 1151.5.

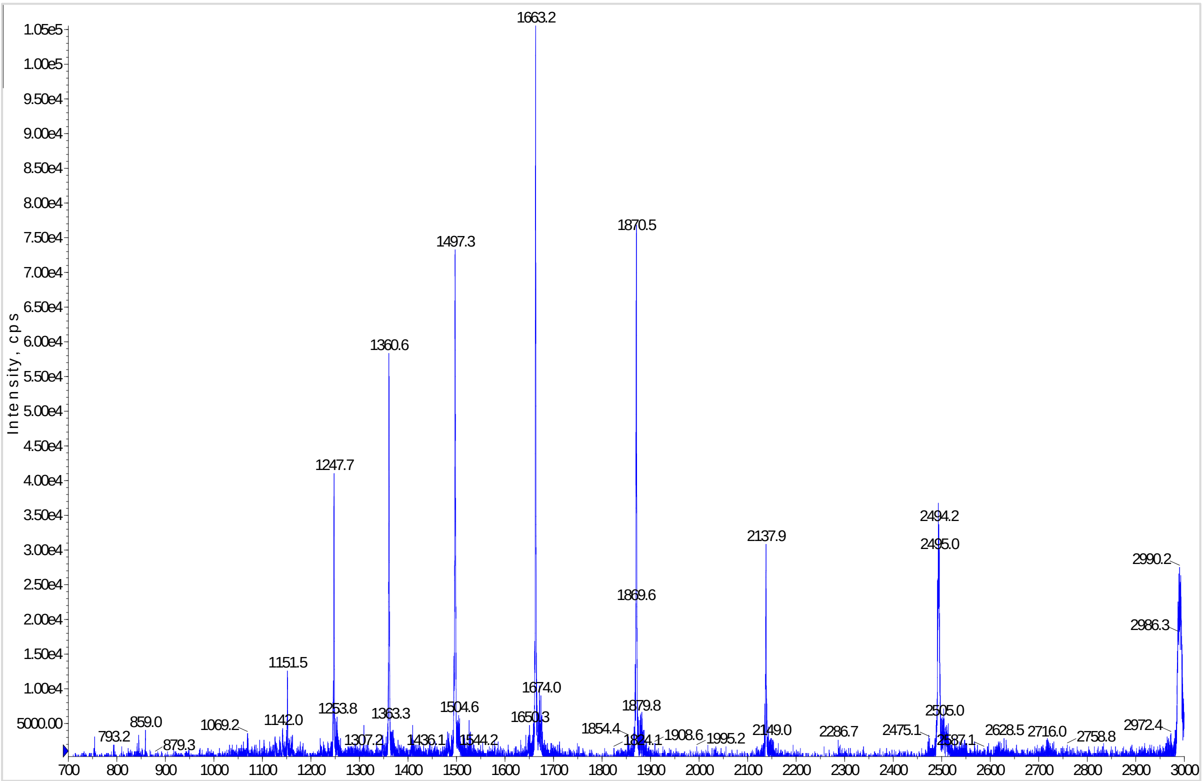

Analytical RP-HPLC chromatograms of Vaccine Candidate 12

Blue trace = % solvent B; 5 min at 0%, 0-20% over 5 min, 20-60% over 30 min.

t_R = 31.85 min

UV detection (λ = 214 nm)

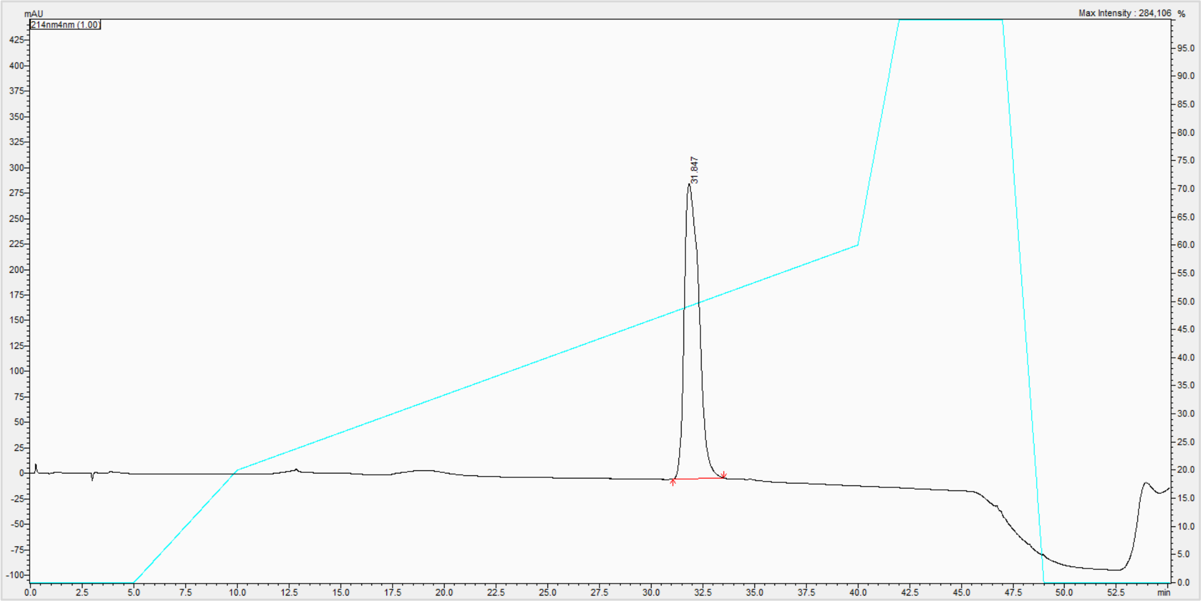

ELSD detection

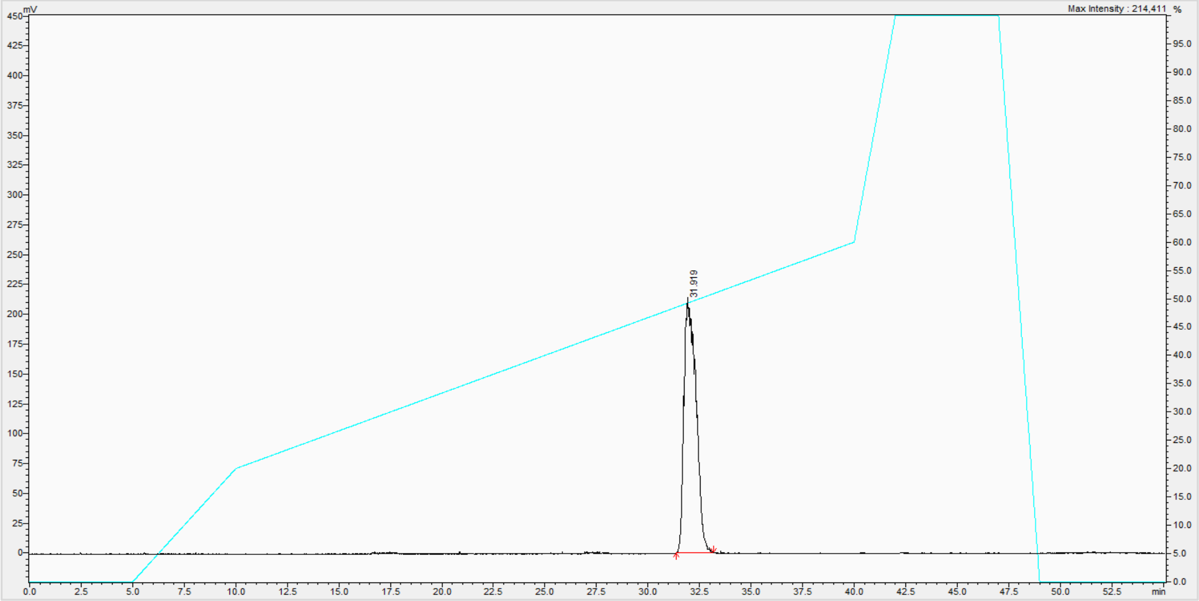

Mass Spectrum of Vaccine Candidate 12

Molecular Weight: 16137.7

MS-ESI (*m/z*): $[M + 9H]^{9+}$ calcd 1794.1, found 1794.9; $[M + 10H]^{10+}$ calcd 1614.8, found 1614.9; $[M + 11H]^{11+}$ calcd 1468.1, found 1468.5; $[M + 12H]^{12+}$ calcd 1345.8, found 1346.3; $[M + 13H]^{13+}$ calcd 1242.4, found 1243.1; $[M + 14H]^{14+}$ calcd 1153.7, found 1154.2; $[M + 15H]^{15+}$ calcd 1076.8, found 1077.3; $[M + 16H]^{16+}$ calcd 1009.6, found 1010.2; $[M + 17H]^{17+}$ calcd 950.3, found 950.5.

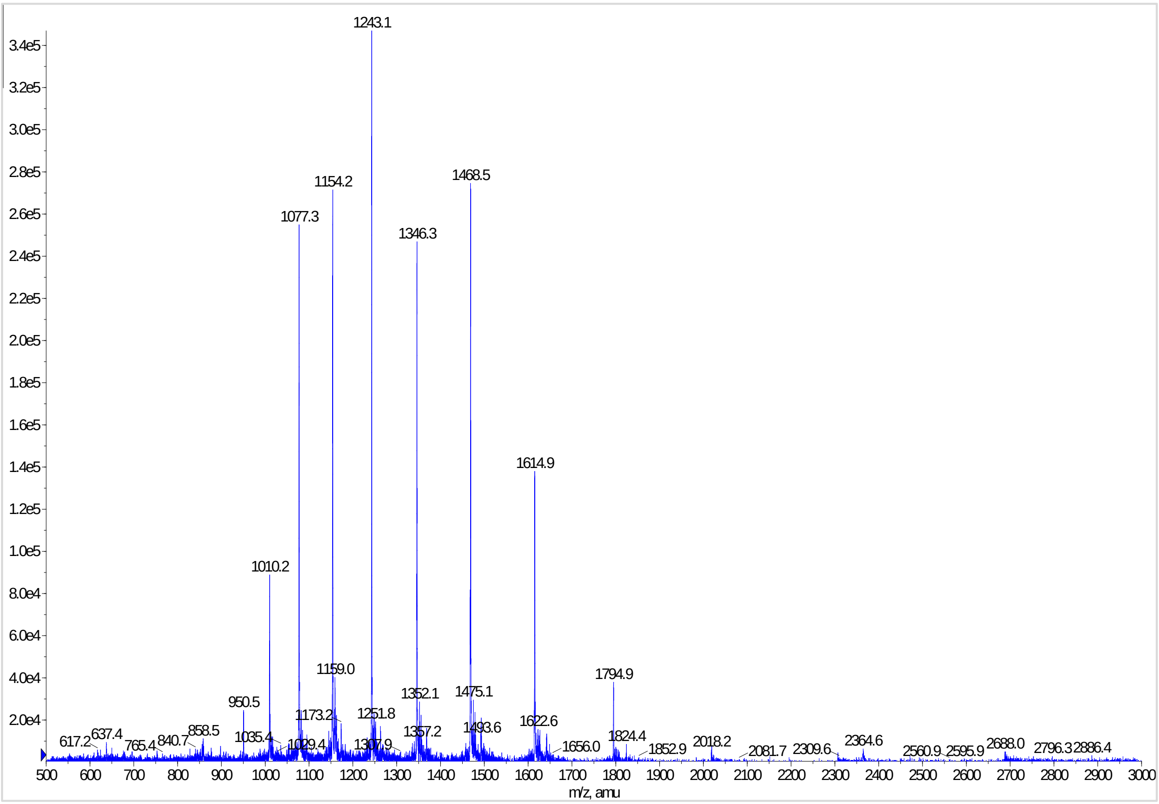

References

1. Duffy, M. G.; Grayson, D. H. *J. Chem. Soc., Perkin Trans. 1* **2002**, 1555-1563.
2. In *Carbohydrates*, 1st ed.; Osborn, H. M. I., Ed. Academic Press: 2003; pp 77-78.
3. Pakulski, Z. *Synthesis* **2003,** *13*, 2074-2078.
4. Zhang, L.; Robertson, C. R.; Green, B. R.; Pruess, T. H.; White, H. S.; Bulaj, G. *J. Med. Chem.* **2009,** *52*, 1310-1316.
5. Brabez, N.; Lynch, R. M.; Xu, L.; Gillies, R. J.; Chassaing, G.; Lavielle, S.; Hruby, V. J. *J. Med. Chem.* **2011,** *54*, 7375-7384.
